# Cancer-Associated Microbiota: From Mechanisms of Disease Causation to Microbiota-Centric Anti-Cancer Approaches

**DOI:** 10.3390/biology11050757

**Published:** 2022-05-16

**Authors:** Priyankar Dey, Saumya Ray Chaudhuri

**Affiliations:** 1 Department of Biotechnology, Thapar Institute of Engineering and Technology, Patiala 147004, India; 2 Council of Scientific and Industrial Research (CSIR), Institute of Microbial Technology, Chandigarh 160036, India; saumya@rocketmail.com

**Keywords:** cancer, commensal bacteria, fecal microbiota transplantation, immune system, inflammation, microbiome, microbiota, pathobiont, probiotics

## Abstract

**Simple Summary:**

Tissue-resident microbiota has been attributed to the overall human health and disease. This comprehensive review summarizes the current understanding of how tissue-resident microbiota is associated with cancer initiation and progression. This review provides a holistic understanding of the microbial mechanisms that can trigger cancer, enlists predominant bacteria that are often associated with cancers, describes microbiota-immune crosstalk, and finally, describes microbiota-centric therapeutic/prophylactic strategies against cancer. Collectively, the current review provides the most comprehensive understanding of the role of tissue-resident microbiota in cancer: from mechanism of disease causation to microbiota-centric anti-cancer approaches.

**Abstract:**

*Helicobacter pylori* infection is the only well-established bacterial cause of cancer. However, due to the integral role of tissue-resident commensals in maintaining tissue-specific immunometabolic homeostasis, accumulated evidence suggests that an imbalance of tissue-resident microbiota that are otherwise considered as commensals, can also promote various types of cancers. Therefore, the present review discusses compelling evidence linking tissue-resident microbiota (especially gut bacteria) with cancer initiation and progression. Experimental evidence supporting the cancer-causing role of gut commensal through the modulation of host-specific processes (e.g., bile acid metabolism, hormonal effects) or by direct DNA damage and toxicity has been discussed. The opportunistic role of commensal through pathoadaptive mutation and overcoming colonization resistance is discussed, and how chronic inflammation triggered by microbiota could be an intermediate in cancer-causing infections has been discussed. Finally, we discuss microbiota-centric strategies, including fecal microbiota transplantation, proven to be beneficial in preventing and treating cancers. Collectively, this review provides a comprehensive understanding of the role of tissue-resident microbiota, their cancer-promoting potentials, and how beneficial bacteria can be used against cancers.

## 1. Introduction

The textbook definition of cancer is a genetic condition arising due to activated proto-oncogene and deactivated tumor-suppressor genes, leading to uncontrolled cell proliferation. Cancer initiation has historically been attributed to chemical and environmental carcinogens, and only a few infectious biological agents are recognized to cause cancers. However, with a better understanding of tissue-specific microbiota (especially in the gut) in human health and disease [1], the role of microbiota in the pathogenesis and progression of various types of cancer through the modulation of genetic and metabolic processes is increasingly getting evident. It is also benignly recognized that tissue-resident microbes can trigger cancer development [2,3], increase the risk and susceptibility of cancer [4,5], modulate cancer-associated immune response [6,7], and improve the efficacy of anti-cancer treatments [8,9].

The majority of infection-associated cancers have a viral origin. Cancers caused by human papillomavirus (anogenital carcinomas, oropharyngeal carcinoma), Epstein–Barr virus (lymphomas, nasopharyngeal carcinoma), human immunodeficiency virus (lymphomas, Kaposi’s sarcoma), hepatitis B and C viruses (hepatocellular carcinoma, lymphoma), human T-cell lymphotropic virus type 1 (adult T-cell leukemia/lymphoma), human herpesvirus 8 (Kaposi’s sarcoma), and Merkel cell polyomavirus (skin cancer) are well documented [10]. Nevertheless, bacterial infections are common among cancer patients due to their immunocompromised nature resulting from malnutrition, chemotherapy, radiation, invasive procedures, and prolonged hospitalization episodes [11]. Persistent infections are typically responsible for 10–20% of all malignancies [12]. Several cases of opportunistic infections caused by tissue-resident commensal bacteria have been reported in cancer patients [13]. The disease causative mechanism of these microbes includes, but is not limited to, the release of antimicrobial peptides from the inflamed mucosa which limit the growth of commensals, but promote the overgrowth of opportunistic pathogens; the loss of crucial commensal species that provide colonization resistance and thereby increase pathogenic infections; improved preferred nutrient availability for the pathogens as a result of depletion of competition with commensals; improved aerobic microenvironment which promotes depletion of anaerobic commensals and overgrowth of facultative pathogens; and the availability of surface adhesion sites promoting better adhesion and colonization for the pathobionts [14]. A detailed discussion regarding these mechanisms has been done in subsequent sections.

An emerging phenotype in cancer patients is the predominance of small intestinal bacteria overgrowth (SIBO) [15], which has earlier been associated with several other chronic conditions [16]. Although no specific type of bacteria is attributed to SIBO, it is mostly associated with the bloom of gut commensals that are otherwise known to exert health-beneficial effects [16]. The exact mechanism through which tissue-resident microbes can trigger cancer is, although underexplored, primarily attributed to eliciting an inflammatory response, which eventually triggers a tumor-promoting environment and the loss of the tissue barrier facilitating the translocation of bacterial metabolites. In fact, due to their systemic effects, gut microbes have also been attributed to exerting cancer-promoting activities on the skin and distant organs such as the brain. Tissue-resident microbes possess a delicate and dynamic interaction with the host. From the host-side, these interactions at the gut are dictated by tissue-specific factors such as pH, pO_2_, the presence of antimicrobials, mucin, bile acid, etc. [1], while from the microbial-side, essential factors include microbial diversity, the availability of nutrients, inter-species competition, and adaptations [17]. Therefore, as discussed in various sections of the manuscript, changes in the tissue architecture and biochemistry are reflected through the specific alterations of tissue-resident microbial profiles in cancer patients [18,19,20].

Although the majority of cancer-microbiota associations in the population-based studies are correlation-based, the role of microbiota as a cancer-promoting agent has been shown experimentally. For example, the transplantation of fecal samples from patients with colorectal cancer to germ-free mice and conventional mice undergoing carcinogenic treatment results in increased polyps, intestinal dysplasia, and inflammation [21]. These observations substantiate the fact that the cancer phenotype is transferable through microbiota and patients with colorectal cancer harbor microbiota that support carcinogenesis. Furthermore, the disruption of gut microbiota can also increase the risk of cancer progression as population-based studies report a higher risk of cancers associated with early-life or prolonged antibiotic treatment [22,23]. Although gut microbiota at higher taxa consist of only a few specific populations, the inter-personal variation and microbial diversity at lower taxonomic levels due to diet, environmental factors, and host genetics may dictate the risk of chronic diseases like cancer [1].

Therefore, the current review centers around the notion that tissue-specific commensal bacteria that are otherwise considered beneficial symbionts under suitable conditions also hold the potential to trigger cancer. Since the distinction between ‘eubiosis’ and ‘dysbiosis’ in terms of microbial populations remains highly liquid, this review summarizes the mechanisms through which commensals to pathobiont transformation of tissue-resident bacteria can trigger cancer. Finally, microbiota-centric anti-cancer approaches, including fecal microbiota transplantation (FMT), have also been discussed in light of improving the efficiency of currently available chemotherapeutic agents. Collectively, this review provides a comprehensive understanding of the role of tissue-resident bacteria as possible cancer-causing agents, and how favorable modulation of microbiota can be used as prophylactic/therapeutic approaches against cancers.

## 2. Epidemiology of Infection-Associated Cancers

Pathogenic infections by bacteria and viruses constitute a significant cause of mortality and morbidity in cancer patients. Especially in patients with underlying hematological malignancies, autopsy data suggest that 60% of deaths are related to infections [24]. These infections include human papillomaviruses (HPV) infections in patients with cervical and oropharyngeal cancer; *Helicobacter pylori* infection in gastric cancer patients; hepatitis B and C viruses infection in patients with hepatocellular carcinoma; Epstein–Barr virus (EBV) infection during Hodgkin’s and non-Hodgkin’s lymphoma and nasopharyngeal carcinoma; and human herpesvirus 8 (HHV8) or Kaposi sarcoma-associated herpesvirus (KSHV) infection in patients with Kaposi sarcoma [25,26,27,28]. *H. pylori*, HPV, HBV, and HCV account for 90% of all cases of infection-related cancers [29]. Although such infection–cancer associations are often closely observed, the infection–cancer type pair is not universal. For instance, *H. pylori* can cause various other gastrointestinal (GI) cancers rather than causing stomach cancer, whereas HPV can also result in non-oropharyngeal head and neck cancers rather than causing vaginal and anal cancers.

Epidemiological data suggest that 2.2 million new cancer cases in 2018 were attributed to infectious origin, where 810,000 instances were attributed to *H. pylori*, 690,000 to HPV, and 360,000 to HBV [30]. Although the overall infection-associated cancer burden was equal in both sexes, the infectious agent spectrum and cancer frequency were highly variable. Women were globally more susceptible to HPV-associated cancers; however, cancers caused by other infectious agents were more common in men [30]. The age-standardized incidence rate (ASIR) of *H. pylori* and HPV-associated cancers was higher in countries like Russia and China compared to India and the USA. ASIR of HBV was higher in China compared to the USA, whereas the opposite trend was seen in the case of HCV. Infection-associated ASIR was highest in eastern Asia (~40 cases per 100,000 person-years), followed by sub-Saharan Africa (~33 cases per 100,000 person–years). In contrast, the number was lowest in northern Europe (13.6 cases per 100,000 person–years) [30]. Data from the 1980s–2000s indicate that 10% of cancer cases in the US, 10–20% in the UK, 3.6% in France, and 29% in China are related to chronic infections [31]. A significant difference in the incidence of infection-associated cancer has been identified between developed (7.7%) and developing (26.3%) countries [32], which could be attributed to factors such as socio-economic conditions, personal and social hygiene, and the availability of preventive and therapeutic means.

Bacterial infections, especially from multi-drug resistant bacteria, are predominant among cancer patients. An earlier systemic analysis of cancer-associated bacteremia indicates that *S. aureus* infection is common in 12% of cancer patients [33]. In patients with nonneutropenic cancers from Asian countries, *S. aureus* infection accounts for ~27% of skin and soft tissue infections and 25% of cases of pneumonia [34]. *Streptococcus* and *Enterococci* are the other predominant bacteremia-causing Gram-positive microbes associated with various cancers. In cancer patients in Italy, Gram-positive and -negative infections account for 33% and 57% of bacteremia, respectively [35]. Cancer patients in Japan have most episodes of bacteremia associated with *Pseudomonas aeruginosa* (14.7%), *E. coli* (18.6%), and *Staphylococcus* spp. (33%) [36]. *E. coli* isolates from cancer patients in the USA are resistant to piperacillin-tazobactam, meropenem, cefepime, amikacin, gentamicin, and ciprofloxacin [37,38]. A recent study indicated that more than 70% of elderly cancer patients suffer from bacteremia from abdominal sources compared to other endogenous or catheter-related infections [39]. Elderly patients harbor increased episodes of infections due to multi-drug-resistant bacteria and a higher infection-related mortality rate.

Opportunistic infection-associated cancer incidence has also been studied from a population perspective. One study reported that, in Texas (USA), preventable oncogenic infections contribute to cancer incidence and may disproportionately impact racial/ethnic minority populations [40]. Specifically, the Hispanic population bears the highest proportions (5.6%) of oncogenic cancers followed by non-Hispanic Black (NHB, 5.4%) and non-Hispanic White (NHW, 2.3%) adults. Hispanics had the largest proportions of all cancers caused by human papillomavirus (HPV, 2.6%) and *Helicobacter pylori* (1.1%), while NHB adults had the highest proportions of all cancers caused by hepatitis C virus (HCV, 1.7%) infection, hepatitis B virus (HBV, 0.7%) infection, and human herpesvirus 8 infection (0.7%). Others have reported that in the USA, NHB had a colorectal cancer (CRC) incidence rate of 49.2 per 100,000, which was higher than NHW (40.2 per 100,000) and Asian/Pacific Islander populations (32.2 per 100,000) [41]. Socioeconomic inequalities account for a large amount of the variation in CRC incidence between racial/ethnic groups, and these socioeconomic differences have been linked to increased obesity, poor diets, and smoking rates [42,43]. Furthermore, inequalities in screening rates and availability [44], as well as healthcare access and follow-up care following aberrant screening findings [45,46,47], can explain the mortality disparities between racial groups. Since diet, lifestyle, and cultural choices are associated with altered gut microbiota and disease risk [48,49], the cancer susceptibility of specific populations is perhaps related to these factors. Although emerging reports support this view [41,50], but large-scale and multi-population-based studies are required to establish the association between microbiota-related cancers with racial, genetic, habitual, cultural, socioeconomical, and dietary patterns of different populations.

## 3. Commensal Overgrowth in Cancer Patients

Changes in bacterial abundance are commonly associated with disease pathogenesis, where the depletion of gut commensals is often associated with increased disease risk. *Bifidobacterium* and *Lactobacillus* spp. are widely considered gut commensal due to their health beneficial effects (e.g., the reduction of mucosal inflammation, improving gut barrier function, short-chain fatty acid (SCFA) production). However, the overgrowth of *Bifidobacterium* has been associated with various case reports related to necrotizing pancreatitis, sepsis, epidural abscess, fatal pulmonary infection, dental caries, and mixed pyogenic infections [51,52]. Similarly, the overgrowth of *Lactobacillus* spp. has also been associated with meningitis, empyema, urinary infection, vaginosis, hepatic abscess, endocarditis, bacteremia, and underlying conditions such as cancer and diabetes [53,54]. Indeed, a recent study reviewing 20 case-controlled reports of gastric microbiota in patients with gastric cancer (GC) has indicated the overgrowth of *Lactobacillus* in GC patients [13]. The emerging cases of infection-associated probiotic microbial overgrowth have been interpreted in diverse manners. For example, one study evaluated gut microbiota composition in children with rotavirus and norovirus infection, and children having infection with enteroaggregative *E*. *coli* and enteropathogenic *E*. *coli* (EPEC). Data showed a lower abundance of Bacteroides, while the increased richness of *Bifidobacteriaceae* in children with bacterial–viral mixed infections was observed [55]. It was emphasized that increased *Bifidobacterium* was likely associated with innate probiotic-protective effects against the infection and the increased abundance was a proportional defense mechanism of the bacteria against the severity of the infection. *Clostridium* spp. of bacteria belonging to the phylum Firmicutes has been associated with diverse GI functions related to human health and disease. A retrospective study including the blood culture records of 40 cancer patients indicated the prevalence of *Clostridium* spp. arising from hepatobiliary tract infection, liver abscess, and bacteremia/enteritis, due to GI tumor in 32% of patients [56]. The most common types of cancer in the cohort were colorectal cancer, pancreatic, and gastric cancer, with a dominant presence of *C. perfringens* and *C. ramnosum*. In line with the evidence that only 50% of gastric cancer patients harbor *H. pylori*, and that the depletion of its population is observed in gastric cancer patients, one study comprising 11 gastric cancer patients, 9 patients with gastritis, and 7 patients with intestinal metaplasia postulated the overgrowth of *Clostridium*, *Lactobacillus*, and *Fusobacterium* as gastric cancer-specific microbial signatures [57]. Furthermore, *Clostridium colicanis* and *Fusobacterium nucleatum* were identified as microbial diagnostic markers for gastric cancers.

Infections involving or originating from the digestive system are more common in cancer patients than in non-cancer individuals [58]. Such infections often involve the overgrowth of *Escherichia coli*, *Klebsiella* spp., and *Enterococcus*, *Viridans streptococci* in the intestine. This is in stark contrast with general hospitals, where *Staphylococcus aureus*, which prefers to colonize the skin, is the most prevalent infection detected [59]. Bloodstream infections are common in cancer patients. An observational study conducted in Mexico, including >4000 cancer patients, indicates 73% of infections are caused by Gram-negative bacteria [60]. The major antibiotic-resistant bacteria in the cancer patients were *E. coli*, *Klebsiella*, *Enterobacter*, *Acinetobacter*, *Pseudomonas aeruginosa*, *Staphylococcus aureus*, and *Enterococcus faecium*. Using 2393 blood cultures, another study from Iran reported a predominance (64%) of Gram-negative bacterial infection in cancer patients [61]. Among the drug-resistant bacteria, *Acinetobacter*, *Pseudomonas*, *Enterobacter*, *E. coli*, and *K. pneumoniae* were the most common ones.

Despite the overgrowth of commensals, multiple clinical reports have shown a decrease in overall microbial diversity, indicating the importance of a rich microbial population in limiting infection and subsequent oncogenesis. For instance, when compared to healthy subjects, HCV-infected patients show significantly decreased Shannon diversity index (α-diversity) and operational taxonomic units (I), in addition to having an overgrowth of *Lactobacillus*, especially in HCV-infected patients with hepatocellular carcinoma (HCC) [62]. Patients with chronic HBV infection without liver cirrhosis or cancer do not have lower microbial diversity [63]. Another study using 419 fecal samples from HCC confirmed patients reported markedly higher α-diversity in HCC patients than in cirrhosis patients, but the overall diversity in HCC patients was lower than in healthy controls [64]. Others have reported that lower microbial diversity is related to a lower response to chemoradiation in cervical cancer patients [65]. A link between high microbial diversity, enhanced tumor infiltration of CD4+ lymphocytes, and CD4 cell activation during radiation therapy was observed. Similar decreased microbial diversity has been reported in patients with head and neck, liver, and stomach cancer [66], colon cancer [67], and breast cancer [68] patient.

## 4. Disrupted Microbiota in Cancer

Several clinical reports show the alterations of diversity and richness, population abundance, and metabolic functions of normal gut microflora associated with a spectrum of cancers. Examination of microbial signatures in the whole-genome and whole-transcriptome from The Cancer Genome Atlas (comprising 33 types of cancers; >18,000 samples) revealed unique microbial signatures in the blood and tissue samples in the largest variety of cancers. These signatures are not only predictive of cancers but also can discriminate between healthy and diseased individuals based on microbial signatures [69]. Another recent study has screened more than 1000 tumor samples from seven different solid tumors and compared the intra-tumor microbial population against more than 500 normal non-cancerous tissue samples [70]. Data show that the tumor-associated microbes and bacterial LPS were mostly intracellular of both cancer and immune cells. The breast cancer tissue harbored a microbiome that was highly diverse compared to cancers from other tissue origins. Specifically, tissues from bone (*Sphingomonas yanoikuyae*), ovary (*Sphingomonas US_602*, *Roseomonas mucosa*), pancreatic (*Enterobacter asburiae*, *Klebsiella pneumoniae*, *Citrobacter freundii*), melanoma (*Paracoccus marcusii*), lung (*Corynebacterium US_1715*) and breast (*Streptococcus infantis*, *Lactobacillus iners*) cancer samples were enriched with specific bacterial populations.

A decrease in microbial α-diversity, an indicator of ‘dysbiosis’ [1], is frequently observed in patients with infection-associated cancers. Compared to healthy individuals, lower microbial diversity has been reported in patients with pulmonary tuberculosis, acquired immunodeficiency syndrome, viral hepatitis, and *Clostridium difficile* infection [63]. Infection-associated alterations in gut microbiota are linked with inflammatory tissue damage due to eliciting pathogen-associated molecular patterns-induced immune responses. Clinical evidence of the close association between different types of cancers and certain bacteria has been identified, like in the case of breast cancer (*Pseudomonas aeruginosa*), thyroid cancer (*Neisseria*, *Streptococcus*), bladder cancer (*Bacteroides fragilis*, *Clostridium cluster I*), colorectal cancer (*Bifidobacteria*, *Porphyromonas*, *Helicobacter*, *Bacteroides*, *Fusobacterium nucleatum*), liver cancer (Proteobacteria), lung cancer (*Enterococcus*, *Streptococcus*, *Prevotella*), stomach and mucosa-associated lymphoid tissue lymphoma (*H. pylori*), pancreatic cancer (Proteobacteria, Bacteroidetes, Firmicutes), hematologic malignancies (*Streptococci*, *Enterococci faecium*), gallbladder cancer (*Salmonella* spp.). Overgrowth of generally considered commensal bacteria, viz., SIBO, is frequently observed in cancer. Although it is not clear whether the overgrowth of commensal could trigger cancer or altered tissue environment during cancer promotes increased colonization of certain bacteria, patients with esophageal, gastric, liver, pancreatic cancer, and cholangiocarcinoma had a SIBO-positive rate of 47.1%, 49.4%, 76.5%, 63.3%, 46.7%, respectively [15]. This indicates that patients with digestive disorders are highly susceptible to SIBO.

## 5. Association of Tissue-Resident Microbiota with Different Types of Cancer

### 5.1. Colorectal Cancer

The gut microbial effects on the risk of development of colorectal cancer have been studied extensively, perhaps more than any other type of cancer, since the hypoxic colon harbors the highest microbial population in the whole body. A plethora of studies has reported that gut microbes can influence colorectal cancer development by mechanisms involving increasing tumor multiplicity and recruitment of tumor-promoting myeloid cell populations, activation of inflammatory transcription factors (e.g., NFκB, STAT3), and increased secretion of pro-inflammatory mediators (e.g., TNFα, IL-1β, IL6) and DNA damage due to oxidative injury [71].

A comparative study between 46 Chinese colorectal cancer (CRC) patients (42–77 y) with 56 healthy volunteers (40–54 y) has shown a significant decrease in *Faecalibacterium*, *Blautia*, *Clostridium*, *Bifidobacterium*, and *Roseburia*, and an increase in *Bacteroides fragilis*, *Fusobacterium nucleatum*, *Enterococcaceae* or *Campylobacter*, *Peptostreptococus*, *Enterococcus faecalis*, *Escherichia coli*, *Shigella* and *Streptococcus gallolyticus* in cancer patients [18]. Further, depletion of butyrate-producing bacteria was associated with cancer patients, but no causative microbe-CRC association was established in the study. Although the direct association between specific microbiota and colon cancer is not well established, environmental factors (e.g., poor diet, physical inactivity, smoking) may trigger CRC by modulating gut microbiota. Indeed, it has been demonstrated that prolonged consumption of a calorie-rich diet can induce intestinal carcinogenesis by altering gut bacteria [72]. Further, the ‘α-bug hypothesis’ suggests that certain oncogenic enterotoxigenic bacteria (e.g., *Bacteroides fragilis*) can directly interact with intestinal epithelial cells to trigger cancer [2]. Others indicated that bacterial metabolites could also trigger cancer, such as sulfidogenic bacteria (e.g., *Fusobacterium*, *Desulfovibrio*, *Bilophila wadsworthia*), which produce hydrogen sulfide, remains strongly associated with colorectal cancer incidences [73].

One of the microbiota-centric risk factors for CRC patients is the opportunistic colonization of *Clostridium difficile*, which can aggravate mucosal inflammation and injury. Indeed, data show that 60% of Iranian patients (mean age of 58 y) with CRC can harbor *C. difficile* infection [74]. Due to radiation and chemotherapy, which dampen normal immune response and depletes normal gut microbiota, cancer patients are at higher risk of accruing *C. difficile* infection, which is associated with prolonged hospitalization and higher mortality rates among CRC patients in the US [75]. Due to frequent reports of increased abundance of specific gut bacteria (*Bacteroides fragilis*, *Fusobacterium nucleatum*, *Porphyromonas asaccharolytica*, *Parvimonas micra*, *Prevotella intermedia*, *Alistipes finegoldii*, *Thermanaerovibrio acidaminovorans*), these could potentially be used as diagnostic markers or risk factors related to colorectal cancer [4]. The majority of the aforementioned studies deal with the microbial effects on cancer initiation and progression. However, one study from China has shown how cancer-associated mutation in the host can alter the tissue-specific microbial population. Using samples from 177 CRC patients, the effects of Kirsten ras (KRAS) mutation on gut microbial patterns were studied [76]. Data showed an increased abundance of *Roseburia*, *Parabacteroides*, *Metascardovia*, *Staphylococcus*, *Staphylococcaceae*, and *Bacillales* in the cancer patients with KRAS mutation. CRC patients without KRAS mutation had a bloom of *Clostridiales*, *Bacteroidetes*, *Lachnospiraceae*, *Coprococcus*, and *Ruminococcaceae*. Collectively, the findings indicated that due to changes in the composition and abundance of the gut microbiota, the prognosis of CRC patients may differ between the mutation and nonmutation groups.

### 5.2. Liver Cancer

The most common type of liver cancer is hepatocellular carcinoma which remains associated with hepatitis virus infection. However, in recent years, chronic metabolic disease (e.g., obesity, nonalcoholic steatohepatitis) is overtaking viral pathogenesis as the leading cause of HCC. Due to the distinct anatomic positioning, the liver is flooded by extracts from the intestinal lumen through the portal circulation. Gut barrier dysfunction due to altered gut microbial composition facilitates gut-to-systemic translocation of bacterial pyrogenic metabolites (e.g., lipopolysaccharide; LPS). Thus, the liver is the next most highly susceptible organ to gut microbial changes after the intestine. One study from the Jiangsu province of China compared the gut microbial profile of patients with HCC related to HBV (n = 35) vs. HCC not associated with HBV and HCV [77]. Data revealed that the patients with HBV-associated HCC had higher microbial richness than healthy subjects (n = 33) or patients with HCC not-related to viral infection (n = 22). However, patients with HCC not related to hepatitis virus infection harbored an increased proportion of inflammatory bacteria such as *Escherichia-Shigella*, *Enterococcus*, and lower level of gut commensals such as *Faecalibacterium*, *Ruminococcus*, *Ruminoclostridium*, which are primarily attributed to the production of SCFA and are anti-inflammatory in nature. These changes in gut microbiota could be different in various stages of liver disease. For instance, a recent study had characterized the gut microbiota in three different stages of HBV-induced progressive liver disease, viz. chronic hepatitis (n = 21), liver cirrhosis (n = 25), and hepatocellular carcinoma (n = 21) [78]. Data showed that patients with chronic hepatitis had a bloom of *Bacteroides*, *Prevotella*, *Atopobium*, *Veillonella*, and *Alistipes*; patients with liver cirrhosis had an increase of *Bacteroides*, *Akkermansia*, *Atopobium*, *Prevotella*, and *Parabacteroides*; and patients with HCC had higher *Bacteroides*, *Veillonella*, *Phenylobacterium*, and *Synechococcus*. While increased Firmicutes-to-Bacteroidetes ratio (F:B) is considered a biomarker for gut dysbiosis, patients with HBV-associated liver disease had lower F:B. Moreover, these patients also have higher proportions of Proteobacteria, which is considered a marker for inflammatory conditions. Although comprehensive in nature, but a major lacuna of the study was its cross-sectional nature that does not reveal a direct link between microbiome and liver disease development.

### 5.3. Lung Cancer

The lung is populated by a distinct microbial population which is essential for maintaining the immunological balance in the airways. Indeed, data show that asthma patients with airway inflammation have increased colonization of pro-inflammatory *Proteobacteria* in the airways [79]. While analyzed by culture-dependent techniques, *Bacteroidetes* and *Firmicutes* have been identified as the predominant microbes in the healthy lung, with a predominance of commensal genera like *Prevotella*, *Veillonella*, and *Streptococcus* [80]. In patients with lung cancer, distinct shifts of microbiota have been reported, which are likely associated with the risk of disease, disease pathogenesis, and progression. For instance, a study intending to define the role of lung microbiota in lung cancer among non-smoker women from the Xuanwei province of China has reported the predominance of *Granulicatella*, *Abiotrophia*, *Streptococcus* in the sputum of cancer patients [81]. It is noteworthy that that this study was unable to rule out the potential that the observed differences were impacted by reverse causation since the study was based on a case-control study design. Another study intending to characterize the microbiome associated with lung cancer in the Korean population has reported a predominance of Firmicutes, *Veillonella*, and *Megasphaera* in the bronchoalveolar fluid of the cancer patients [82]. However, overall small sample size (n = 28) and no consideration of lung function or antibiotic intake potentially limits the understanding of microbe–cancer association from the results. While exploring the lung tissue-associated microbial signatures in smokers with lung cancer from the US population (n = 40), Liu and colleagues reported that patients with cancer have higher microbial diversity in lung tissue than patients with only emphysema [19]. In this study, since spirometry-based lung function was not distributed between the test groups, it could have affected the microbiota composition variance. Community composition of lung microbiota from 29,133 smokers from Finland showed a lower proportions of the phylum Proteobacteria (especially genus *Acinetobacter* and *Acidovorax*) and increased Firmicutes (*Streptococcus*) and Bacteroidetes (*Prevotella*) in lung cancer patients. Although these microbe–cancer associations are primarily correlative in nature, *Mycobacterium tuberculosis* infection is a distinct risk factor for lung cancer [83].

### 5.4. Hematological Cancer

In Thai patients with acute myeloid leukemia with neutropenic fever during intensive chemotherapy, it has been observed that the populations of phylum Firmicutes were predominant during neutropenic fever. In contrast, the abundance of Bacteroidetes and Proteobacteria was higher after bone marrow recovery [84]. During the febrile neutropenia period, *Enterococcus* was more abundant relative to the pretreatment period, while the decline of *Escherichia* was observed during the same period. It was also observed that the OTU richness and microbial diversity were significantly lower during the febrile neutropenic period compared to the pretreatment period. The biggest limitation of this study was the small population size (n = 10) which might have hindered proper data interpretation caused by limited statistical power. Others have demonstrated that the GI microbiota can serve as a distinguishable marker for pediatric and adolescent acute lymphoblastic leukemia (ALL). Using fecal samples from 51 matched pediatric and adolescent patients with ALL and their healthy siblings from the US, it was demonstrated that the ALL patients harbor significantly less microbial diversity while sharing several common microbes at the genus level [85]. The predominance of *Anaerostipes*, *Coprococcus*, *Roseburia*, and *Ruminococcus* was lower in the patients compared to the control. Another study examined the chronological alterations of GI microbes in children from Malaysia with ALL during the start-, during-, and following cessation of chemotherapy [86]. It was observed that compared to healthy controls, higher inter-personal microbial variability was present in children with ALL. The abundance of Bacteroidetes, predominant in children with ALL before chemotherapy, decreased after chemotherapy. Although the microbiota restoration partially occurred after the cessation of chemotherapy, the bacterial β-diversity remained unchanged. A detailed discussion on childhood ALL has been undertaken in a recent review that predominantly indicated Enterococcal infection in ALL patients post-chemotherapy, while long-term loss of gut commensals (e.g., *Faecalibacterium*) has been reported in ALL survivors [87]. Ataxia-telangiectasia (A-T) is a genetic condition characterized by a high incidence of B-cell lymphoma. Small sample size (n = 7 control and n = 7 patient) was the biggest limitation of the study in addition to failure to establish association between altered microbiota post-chemotherapy with the risk of future health complications. Researchers using an experimental A-T mice model and mouse colonies harboring specific bacterial communities have indicated that gut microbiota may contribute to the disease penetrance, lifespan of an animal, molecular oxidative stress, and systemic leukocyte genotoxicity [88]. In particular, an overgrowth of *Lactobacillus johnsonii* was observed in the cancer-resistant mouse colonies, where it could mitigate inflammation and genotoxicity when administered orally. Raising A-T mutant mice in a sterile condition increased life span and lower inflammation and lymphoma penetrance.

### 5.5. Pancreatic Cancer

Pancreatic cancer is one of the leading causes of mortality in developing countries, and clinical data suggest distinct microbial signatures associated with pancreatic cancer patients. Due to the metabolic importance of the pancreas in conditions like diabetes and obesity, the composition of gut microbiota may likely affect pancreatic disease [89]. Indeed, it was observed that when the gut microbial phenotype of 30 pancreatic cancer patients from Israel was compared with that of 13 healthy subjects, the Firmicutes to Bacteroides ratio was found to be lower in the patients, in addition to a reduction of *Bacteroidales*, *Odoribacter*, *Lachnospiraceae UG_008*, *Veronella*, *Megasphaera* and *Akkermansia* in the patients [89]. On the other hand, healthy individuals had an increased population of *Clostridiales*, *Anerostripes*, *Ruminococcaceae*, *Faecalibacterium*, and *Subdoligranulum*, compared to pancreatic cancer patients. A limitation of this study was small population size that not only resulted in limited statistical power, but also required to enroll subjects with pre-cancerous lesions or very early-stage which would have provided better understanding of the microbiota-disease association. In a case-control study from the US, researchers had compared the bacterial and fungal population of duodenal fluid between patients with pancreas ductal adenocarcinoma (PDAC; n = 74) and healthy controls (n = 134) [90]. The populations of *Fusobacterium*, *Enterococcus*, *Lactobacillaceae*, and *Bifidobacterium* were higher in patients with PDAC compared to controls. The study had several limitations, including (a) patient cohort from a single medical facility, (b) presence of confounding variables between patients and controls, and (c) no inclusion of the pathophysiological effects of pancreatic cancer, which possibly affects the microbiota.

In the case of PDAC, it is interesting that several studies have indicated the oral microbial population as a marker for cancer and have been reviewed elsewhere [91]. One such study using Chinese population intending to identify the changes in the oral bacterial community in the tongue coating of 30 patients with pancreatic head carcinoma reported that the patients had increased microbial diversity in addition to a distinct increase in the populations of *Leptotrichia*, *Fusobacterium*, *Rothia*, *Actinomyces*, *Corynebacterium*, *Atopobium*, *Peptostreptococcus*, *Catonella*, *Oribacterium*, *Filifactor*, *Campylobacter*, *Moraxella* and *Tannerella* were predominant in the patients compared to healthy controls [92]. Similar observations of altered oral microbiota were observed in another study from Iran, where 273 patients with pancreatic adenocarcinoma had an increased population of *Enterobacteriaceae*, *Lachnospiraceae*, *Bacteroidaceae*, and *Staphylococcaceae*. At the same time, *Haemophilus* was decreased in patients with pancreatic cancer [93]. Although the population size was large enough, but the study suffered from two major limitations: (a) The researchers were unable to address potential confounding by these factors since no information on oral health was gathered, and (b) because saliva samples were taken from the cancer patients at the time of diagnosis, it was not possible to determine if any microbiota–disease relationships were linked to the genesis or existence of the illness.

### 5.6. Breast Cancer

Breast cancer is one of the leading types of cancer, and the role of microbiota in the pathogenesis of breast cancer has been identified. Using fecal samples from 32 breast cancer patients in the USA, others have reported that the abundances of *Bifidobacterium*, *Blautia*, *Faecalibacterium prausnitzii*, and *Blautia* were different at different stages of breast cancer; especially, the abundance of *Balutia* was higher in grade III cancer patients relative to grade I patients. Moreover, in line with the obesity-cancer relationship, it was observed that the total microbial count and populations of *F. prausnitzii*, *Firmicutes*, *Blautia*, and *Egerthella* were elevated in cancer patients with increased BMI. Using fecal samples from 48 postmenopausal women with breast cancer, an estrogen-independent lowering of microbial diversity was reported in breast cancer patients [68]. Additionally, increased populations of *Clostridiaceae*, *Faecalibacterium*, and *Ruminococcaceae* and lower populations of *Dorea* and *Lachnospiraceae* were observed in the cancer patients. Limitations of the study include small sample size, which precluded the assessment of minor taxa and of interactions between microbiota metrics and known risk factors, particularly estrogens. Apart from fecal microbiota, which essentially represents the gut microbial population, samples from breast tissue of patients from the US also indicate an altered microbial profile associated with cancer. For instance, using breast tumor tissue and paired normal adjacent tissue from the same patient, one study had reported that *Proteobacteria*, *Firmicutes*, *Actinobacteria*, *Bacteroidetes*, and *Verrucomicrobia* are the most predominant bacterial genus across the samples [94]. The populations of *Methylobacterium radiotolerans* and *Sphingomonas yanoikuyae* were highly enriched in tumor and normal tissue, respectively. Their relative abundances were inversely correlated with their respective tissue type, indicating a causal relationship between these bacterial populations and breast cancer. Using 668 breast tumor tissue samples and normal adjacent tissue data from The Cancer Genome Atlas, another group of researchers has indicated that Proteobacteria, Actinobacteria, and Firmicutes were the predominant phylum in the breast cancer tissue [95]. *Mycobacterium fortuitum* and *Mycobacterium phlei* were the two most abundant microbe in the cancer tissue.

### 5.7. Oral Cancer

Due to poor oral hygiene, microbial growth and biofilm formation have been attributed to oral cancer. Using tumorous and normal tissue samples from buccal mucosa from 50 Chinese patients (mean age 60.7 y), it was reported that the bacterial richness and diversity were higher. At the same time, the populations of *Prevotellaceae*, *Fusobacteriaceae*, *Flavobacteriaceae*, *Lachnospiraceae*, *Peptostreptococcaceae*, and *Campylobacteraceae* were predominant in cancer patients [20]. *Fusobacterium nucleatum*, *Prevotella intermedia*, *Aggregatibacter segnis*, *Capnocytophaga leadbetteri*, *Peptostreptococcus stomatis* were the most abundant bacteria at the species level. Microbial predicted functional analysis showed that bacterial functions related to chemotaxis, flagellar assembly, and LPS biosynthesis were associated with microbes residing in the cancerous tissue. Data from Hungary have linked *Fusobacterium*, *Veillonella*, *Actinomyces*, *Clostridium*, *Haemophilus*, and *Enterobacteriaceae* with epithelial precursor lesions and oral cancer [96]. In this study intended to compare the oral microbiota in tumor and normal tissue in 21 Hungarian patients with oral squamous cell carcinoma, it was reported that several *Streptococcus* spp., *Peptostreptococcus stomatis*, *Gemella haemolysans*, *Gemella morbillorum*, *Johnsonella ignava*, and *Streptococcus parasanguinis* were the predominant bacteria at the site of the tumor, while the abundance of *Granulicatella adiacens* was highest in the non-tumor tissue. Another study had profiled the oral cancers and anatomically matched contralateral non-tumorous tissue from the same patients (n = 83) from the US, and reported that the population of Firmicutes and Actinobacteria was significantly lower in cancer tissue [97]. *Streptococcus* and *Rothia* were reduced, whereas the population of *Fusobacterium* was higher in cancer patients. The study was mostly observational in nature and was unable to decipher whether the observed microbial reflect the fact that particular bacteria are better suited to attach to and develop in the cancer microenvironment or if they promote cancer. Finally, a comparison of oral microbiota of saliva samples from 60 oral cancer and 80 healthy controls from Japan revealed that the microbial populations in the two cohorts had a difference in overall microbial diversity [98]. *Peptostreptococcus*, *Fusobacterium*, *Alloprevotella*, and *Capnocytophaga* were relatively more abundant in the samples from cancer patients, while the healthy cohort had higher populations of *Rothia* and *Haemophilus*.

### 5.8. Other Types of Cancers

Although less explored, alterations in resident bacterial populations have also been reported in other types of cancers. One group of researchers had compared the microbiota from urinary bladder carcinoma and urine samples from the same patients from Hungary to improve the diagnostic accuracy [99]. It was reported that *Lactobacillus*, *Corynebacterium*, *Streptococcus*, and *Staphylococcus* were highly abundant in the urine sample. In contrast, *Clostridium* sensu stricto, *Akkermansia*, *Bacteroides*, *Enterobacter*, and *Klebsiella dominated the bladder tissue samples*. Limited sample number (n = 10) was the biggest limitation of this study. In another study, using samples from bladder cancer patients from China (patient n = 62 and control n = 16), it was reported that the microbial diversity was higher in patients with recurrent cancer than in non-recurrent ones [5]. *Anoxybacillus*, *Massilia*, *Thermomonas*, *Brachybacterium*, *Micrococcus*, *Nocardioides*, *Larkinella*, *Jeotgalibacillus*, and *Geomicrobium* were highly abundant in patients with recurrent bladder cancer. Limited follow-up period, lack of information about microbial population stability over time, and a possible cross-contamination of samples from adjacent pelvic niches are the drawbacks of the study. In the case of prostate cancer, the populations of *Faecalibacterium prausnitzii*, *Bacteroides massiliensis*, *Mycoplasma genitalium*, and *Eubacterium rectalie* have been associated with the risk of disease development and disease severity [5]. The abundance of *Micrococcus luteus*, *Fusobacterium nucleatum*, *Streptococcus agalactieae*, and *Corynebacterium diphtheriae* were reported to be elevated in the renal cancer samples compared to the healthy counterparts from the US (n = 6; 66% Hispanic) [100]. In 74 Chinese patients with thyroid cancer, an association of the microbiota with disease pathogenesis and thyroid functional index has been drawn. For instance, apart from differences in microbial diversity indexes, in patients with thyroid cancer, the populations of *Neisseria* and *Streptococcus* were found to be higher. In contrast, *Butyricimonas* and *Lactobacillus* were lower than the healthy controls [101]. In renal carcinoma tissue, bacterial overgrowth was evident and increased abundance of *Aeromonas salmonicida*, *Parageobacillus toebii*, *Mesorhizobium cicero*, and *Bacillus cereus* compared to the healthy tissue from Austrian patients (n = 5) [102]. Progressively increased colonization of anaerobes and decreased population of commensal *Lactobacillus* are associated with cervical cancer patients from the US [103].

Collectively, although several similar observational studies indicating the changes in diversity and microbial populations are available, these studies have been criticized due to low sample number, and for primarily representing correlative associations rather than establishing any microbiota–disease causal relationship.

## 6. The Classical Case of *H. pylori* Infection, Gut Microbiota, and Gastric Cancer

Gastric cancer caused by *H. pylori* infection is the first reported and one of the well-studied cases of infection-associated cancer. *H. pylori* is a spiral-shaped Gram-negative bacterium that colonizes the gastric mucosal layer. *H. pylori* has been coined as type I carcinogen and accounts for 5.5% of global infection-related cancer [104]. Specifically, *H. pylori* have been attributed to 60–80% of all gastric cancer cases globally [31]. In the highly acidic gastric environment, *H. pylori* have evolved to survive and colonize by converting urea to ammonia, creating a pH-neutral microenvironment surrounding the bacterium [105]. Although *H. pylori* infect 50% of the global population [106] but do not cause clinical symptoms in most individuals, persistent infection increases the risk of chronic disease, including a 10% risk of peptic ulcer, <3% gastric adenocarcinoma, and <0.1% mucosa-associated lymphoid tissue lymphoma [107]. Epidemiological data suggest that subjects infected with *H. pylori* have an increased risk of gastric cancer [108,109].

Experimental evidence from GI carcinoma-predisposed INS-GAS mice reveals that eradicating *H. pylori* using antibiotic cocktails resulted in a reduction in GI intraepithelial neoplasia and the extent of gastric inflammation, reducing the risk of gastric cancer [110]. Indeed, others have shown that a *H. pylori* monoassociation in germ-free INS-GAS mice resulted in progressive gastritis, epithelial defects, oxyntic atrophy, marked foveolar hyperplasia, dysplasia, and robust serum and tissue proinflammatory immune responses [111]. Certain strains of *H. pylori* code for cytotoxin CagA, which, upon insertion in the gastric mucosal cells, alters the stomach cells’ structure and facilitates colonization of the bacterium [112,113]. The persistent infection triggers chronic inflammation, severe chronic atrophic gastritis, and ulceration, later giving rise to cancer. CagA can bind to the tyrosine phosphatase SHP-2 protein and deregulate phosphatase activity of SHP-2 and trigger the Ras/MAPK-signaling cascade, which is attributed to several types of cancers [114]. Indeed, isolated experiments using CagA-treated gastric organoids also show increased proto-oncogene c-Met expression and cellular proliferation [115].

One of the mechanisms through which *H. pylori* can trigger gastric cancer is by inducing host DNA damage. The expression of activation-induced cytidine deaminase (AID) promotes somatic hypermutation. Data suggest that ‘cag’ pathogenicity island-positive *H. pylori* can trigger aberrant AID expression in gastric cells in a mechanism involving NFκB-dependent inflammation and alerted tumor-suppressive TP53 expression [116]. Mismatch repair (MMR) is crucial for the host cells to prevent cancer initiation from DNA damage-associated events. Gastric epithelial cells infected by *H. pylori* have decreased MMR-associated protein expression through mechanisms involving reduced mRNA levels of repair genes [117]. This enhances the risk of mutation accumulation in gastric mucosa, causing stomach cancer. Specifically, *H. pylori* infection decreases the expression of MutS, MutL, hMSH2, and hMSH6 DNA repair proteins in a dose-dependent manner. In support, others have shown that *H. pylori* infection reduces the expression of several regulatory miRNAs associated with MMR [118]. These data collectively demonstrate how pathogenic insult could aggravate cancer-promoting events in the gastric mucosa.

A distinct microbial profile in patients with *H. pylori* infections has been reported. While normal subjects have Proteobacteria, Firmicutes, Actinobacteria, Bacteroidetes, and Fusobacteria as the predominant phyla, patients with *H. pylori* infection have *H. pylori* as the single most dominant bacterium in the stomach [119]. Using 16S sequencing of gut microbiota from 60 subjects, it was demonstrated that microbiota of uninfected subjects had a lower abundance of *Succinivibrio*, *Coriobacteriaceae*, *Enterococcaceae bacterium* RF39, and *Rikenellaceae* compared to infected patients [120]. Low populations of *H. pylori* were reported in patients with an advanced gastric premalignant lesion; however, patients with gastric cancer had an increased population of *Lactobacillus*, *Streptococcus*, *Veillonella*, and *Prevotella* [121]. In another study, 103 gastric biopsy samples from *H. pylori-positive* patients were analyzed for bacterial populations [122]. From these samples, 201 different bacteria were isolated, among which gastric acid-resistant genera like *Streptococcus*, *Neisseria*, *Rothia*, and *Staphylococcus* were predominant. Especially, Staphylococcus epidermidis, *Staphylococcus warneri*, Staphylococcus capitis, Staphylococcus aureus, *Brevibacterium* spp., and *Klebsiella pneumoniae* were the most abundant bacteria that possess potent urease activity to withstand the strong gastric acidic environment. Another study compared the fecal microbial profile of 212 *H. pylori-infected* patients with 212 normal controls. Apart from having higher α-diversity, patients with *H. pylori* infection harbored more Bacteroidales, Burkholderiales, Holdemanella, and β-proteobacteria compared to the controls [123]. Specifically, microbiota from healthy subjects were dominated by Bacteroides-prevalent microbial enterotype 1, whereas the patients had higher Prevotella-dominant enterotype 2 compared to the controls. A comparative analysis of the altered gut microbial metabolic functions calculated on the background of 16S rRNA data from 37 *H. pylori*-positive and 18 *H. pylori*-negative children showed the enrichment of microbial functions related to energy metabolism, cancer formation, cell growth, glycan biosynthesis and metabolism, and cell motility [124]. These studies collectively shed light on the cancer-promoting mechanisms of *H. pylori* and indicate how *H. pylori* colonization shapes tissue-resident microbiota to promote a microenvironment that favors cancer.

## 7. Microbiota Changes in Patients with Cancer-Causing Viral Infections

Beyond bacteria, patients with chronic infection by cancer-causing viruses also show dramatically altered microbial profiles (Table 1). Although the virus–microbiota–cancer axis remains critically underexplored and the majority of the studies are merely observational in nature, commensal microbiota have been linked to the enhancement of viral replication and the extent of infections [125]. Chronic viral infection can also reduce the population of commensal bacteria, leading to chronic disease. For instance, an ultravirulent mutant phage has been reported to deplete the population of butyrate-producing commensal *Roseburia intestinalis* [126]. The altered virome has also been reported in patients with obesity and diabetes [127]. Since, chronic metabolic diseases are a risk factor for cancer, how the virome can influence cancer-promoting by influencing gut microbiota remains critically underexplored.

## 8. Cancer-Promoting Role of Microbiota

The gut microbial population hosts an overwhelming 3.3 million functional genes with diverse metabolic capacities, which can directly or indirectly dictate disease risk [1]. Obesity is a risk factor for several types of cancer, including HCC. Experimental data show that gut microbiota transfer can be a mode of transfer of non-communicable disease phenotype [137], thus, a mode of transfer of chronic disease risk. Indeed, one of the most compelling pieces of evidence supporting the role of gut microbiota in cancer development comes from a murine experiment that demonstrated that the oral gavage of fecal matter from CRC patients could promote the onset of CRC in both germ-free and conventional mice [21]. Others have shown that germ-free or conventional mice with antibiotic treatment are protected from lung cancer development that is otherwise triggered by *Kras* mutation and the loss of *p53* if lung microbiota is not distorted by antibiotics [138]. Microbial infections are responsible for 20% of carcinogenesis, and microbial commensal imbalance is linked to a greater incidence of cancers [139]. Due to altered gut microbiota or opportunistic infections, pathobionts can multiply and release specific toxins that cause host DNA breakage, contributing to genomic instability and tumor progression.

### 8.1. Microbiota Promotes Cancer by Modulation of Bile Acid Metabolism

Gut microbiota can influence cancer development through bile acids. Saturated fat can promote microbial overgrowth resulting in increased primary to secondary bile acid formation (e.g., deoxycholic acid, lithocholic acid) with cancer-promoting properties. An earlier case-control study indicates a higher level of deoxycholic acid in individuals with colorectal adenomas, while a strong association was established between colonic microbiota-derived deoxycholic and colorectal adenoma [140,141]. The metabolism of animal fat-derived taurine and subsequent formation of taurine-conjugated bile acids by specific gut microbes (e.g., *Bilophila wadsworthia*) favors the microbial generation of hydrogen sulfide and deoxycholic acid, both of which are genotoxic and tumor-promoting agents [142]. *Clostridium*, *Bifidobacterium*, *Lactobacillus*, *Enterococcus*, *Faecalibacterium*, and *Roseburia* can hydrolyze secondary bile acids, thereby regulating secondary bile acid-mediated cell proliferating signaling [1,143]. Increased microbiota-derived secondary bile acids can promote mucosal cell proliferation by various mechanisms involving the activation of epidermal growth factor receptor and extracellular signal-regulated kinase [144] and Farnesoid X receptor [145]. Secondary bile acids can also trigger oxidative damage resulting in DNA instability by disrupting mitosis, defects in spindle assembly checkpoints, abrupt cell-cycle arrest, erroneous chromosome alignment, and multipolar divisions [146].

### 8.2. Microbiota Promotes Cancer by Modulating Hormonal Effects

Gut bacteria can influence host metabolic processes by modulating hormonal effects. Gut commensals can alter the risk of postmenopausal estrogen receptor-positive breast cancer by metabolizing estrogen in females. In contrast, gut microbes can trigger prostate cancer in men by metabolizing glucocorticoids to generate 11-oxyandrogens [104]. The association between bacterial β-glucuronidase and host estrogen hormones has been studied extensively. Due to increase of β-glucuronidase-secreting spp. (e.g., *Clostridium leptum*, *Clostridium coccoides*), estrogen is deconjugated, and thereby more estrogen binds to target cells and induces cell proliferation [147]. As a result, increased estrogen levels are linked to breast cancer risk, which supports the finding that the gut microbial composition of women with breast cancer differs from healthy controls and implies that several β-glucuronidase-producing gut bacteria are likely linked to breast cancer development [148]. A recent study demonstrates that androgen-producing gut bacteria can trigger the onset of prostate cancer and resistance to hormone therapy [149]. The data showed that androgen deprivation in mice and humans results in the bloom of a commensal population, which promotes castration-resistant in mice. Germ-free animals had delayed hormone resistance and slower tumor growth. Additionally, fecal transplantation from hormone-resistant prostate cancer-bearing mice promotes tumor development in mice with low-androgen levels. Therefore, microbial influence on the host system by hormone synthesis may influence the efficacy of hormone therapy in cancer patients.

### 8.3. Cancer Promoting Roles of Bacterial Toxins

Specific bacterial populations can regulate cellular signaling pathways to induce inflammation, which is a critical feature in chronic conditions like cancer [150]. Certain bacterial toxins can also promote tumorigenesis by inducing inflammation. Utilizing high-throughput sequencing techniques, it was demonstrated that inflammation is related to alterations in the gut microbial population in colitis-susceptible interleukin-10-deficient (*Il10*^−/−^) mice and that colonization of the intestine with commensal *Escherichia coli* NC101 triggers invasive carcinoma in *Il10*^−/−^ mice treated with azoxymethane [3]. When the polyketide synthase (pks) genotoxic island, coding for non-ribosomal peptide-type genotoxin colibactin, was genetically removed from the commensal *E. coli*, the extent of tumor multiplicity and invasion was reduced independent of intestinal inflammation. In line, the predominance of pks+ *E. coli* has been reported in CRC patients [151]. Earlier data show that cytotoxic necrotizing factor 1 (CNF) from uropathogenic *E. coli* can activate Rho GTP-binding protein and attenuate apoptosis by affecting mitochondrial homeostasis and upregulating expression of Bcl-2 family proteins [152]. Later, it was shown that CNF can strongly upregulate COX2 in a RhoA-dependent manner [153] and by stimulating the Cdc42-PAK1 axis, it promotes prostate cancer growth [154]. Therefore, prolonged infection and survival of CNF+ *E. coli* can promote tumorigenesis in association with persistent inflammation. Avirulence protein A (AvrA) secreted by *Salmonella typhi* can influence CRC development. Azoxymethane/dextran sulfate sodium-treated mice experimentally infected with AvrA+ *Salmonella*, had increased tumorigenesis compared to mice with AvrA-defficient *Salmonella* infection [155]. AvrA+ *Salmonella* was able to upregulate β-catenin nuclear signaling which is a marker for cancer stem-cell proliferation. *Salmonella* AvrA protein and anti-AvrA antibodies were detected in colonic mucosa of experimentally infected mice and expression of AvrA was reported in human CRC tissue [156]. *Pasteurella multocida* toxin (PMT) possess strong mitogenic potentials. PMT can cause the constitutive expression of cell proliferation- and survival-associated genes of the G-protein family and suppressive apoptosis by the activation of mitochondrial pro-survival pathways [157]. However, clinical evidence linking the prevalence of PMT+ bacteria with cancer is lacking.

It has also been reported that specific bacterial proteins can modulate cell proliferation and survival signaling pathways to trigger tumorigenesis. CagA protein (*H. pylori*), effector adhesin A (*Fusobacterium nucleatum*), and metalloproteinase toxin (*Bacteroides fragilis*) can interact with cell-surface E-cadherin to disrupt intracellular junctions, trigger cell proliferation, and cancerous transformation. The natural killer cell-mediated killing of tumor cells is inhibited in the presence of *Fusobacterium nucleatum*, which is predominant in the tumor microenvironment. These activities are attributed to the Fap2 protein present in *F. nucleatum*, which inhibits NK cell activities by directly interacting with T cell immunoglobulin and ITIM domain, an inhibitory domain in lymphocytes [158]. Indeed, *F. nucleatum* has been reported to promote chemoresistance in CRC patients through loss of tumor-suppressive miR-18a* and miR-4802 by activating TLR4/MyD88-dependent signaling [159]. Enhanced β-catenin signaling in colonic epithelial cells increased the cell proliferation, myeloid cell accumulation, and production of proinflammatory cytokines observed in mice inoculated with *F. nucleatum* [160]. In fact, the 10–100 times increased the expression of FadA adhesion factor of *F. nucleatum* in the CRC patients where FadA expression is proportional to the increased levels of inflammation and cancer-associated genes. *F. nucleatum* also enhances tumor frequency and attracts tumor-infiltrating myeloid cells in the CRC susceptible Apc^Min/+^ mice without affecting colitis or enteritis [161].

### 8.4. Microbiota Cause DNA Damage and Hinders DNA Repair

DNA breakage is one of the critical cellular injuries that potentiate tumorigenesis. Commensal and extraintestinal pathogenic *E. coli* have been identified to induce DNA double-strand breaks [3]. *E. coli* produces colibactin and cytolethal distending toxins, which can induce DNA double-stranded breaks when released adjacent to the intestinal epithelium, triggering temporary cell cycle arrest, triggering genetic mutations, and may lead to tumor formation [162]. Other gut pathobionts can hinder DNA repair mechanisms, leading to cancer progression. *Shigella flexneri* can trigger the degradation of p53, an essential regulator of DNA damage response and repair, thereby increasing the chances of stable mutation and genomic alterations in the host cell [163]. DNA damage can occur due to redox imbalance, and certain gut bacteria have been attributed to such a process. For instance, *Helicobacter pylori* or *Bacteroides fragilis* can contribute to oxidative injury by activating spermine oxidase in the host cells, which can generate hydrogen peroxide and trigger oxidative damage to DNA and cellular biomolecules [164]. Various anaerobic fermentative bacteria belonging to *Lactobacillus* and *Clostridia* spp. generate ethanol. Gut microbe-mediated ethanol to aldehyde production can promote cancer by reacting with deoxyguanosine, forming N_2_-ethylidenedeoxyguanosine adduct and thereby intra-strand crosslinking [165]. EPEC, *Campylobacter*, *Shigella*, and *Haemophilus ducrey* has been reported to cause single- and double-strand DNA breakage, thereby leading to insertion–deletion mutations by causing infidelity of the host DNA repair mechanism [166]. EPEC, often reported in CRC patients, can dysfunctionally host MMR and promote CRC [167]. Data show that the EspF protein secreted by EPEC can deplete MMR proteins through post-transcriptional mechanisms, and by targeting mitochondrial processes. EPEC infection can also elevate oxidative stress, promoting DNA damage and increasing the spontaneous mutation rate. Gut commensal *Enterococcus faecalis* is known to generate extracellular superoxide radical and increase chromosomal instability through macrophage-mediated bystander effects [168]. *E. faecalis*-derived 4-hydroxy-2-nonenal (4-HNE) has been shown to induce cell-cycle arrest and disrupt mitotic spindle formation in colonocytes. Silencing of glutathione S-transferase increased 4-HNE-mediated genotoxicity. IL-10 KO mice developed CRC only colonized with superoxide-producing E. faecalis but not with colonization with superoxide-deficient *E. faecalis*.

### 8.5. Microbiota Influence Chemotherapeutic Efficiency

Tissue-resident microbes can influence cancer by affecting anti-cancer treatment. For example, tumor-beading germ-free mice or mice treated with antibiotics to deplete microbiota, demonstrate increased resistance to the chemotherapeutic drug cyclophosphamide [169]. The increasing multidrug resistance in cancer patients has been attributed to the diverse metabolic capacities of the microbiota [170]. In line, data show that 76% of patients with pancreatic ductal adenocarcinoma have a higher population of γ-proteobacteria in the tumor tissue [171]. Intratumor γ-proteobacteria can metabolize the chemotherapeutic drug gemcitabine (2′,2′-difluorodeoxycytidine) to its inactive form, 2′,2′-difluorodeoxyuridine through the enzymatic action of cytidine deaminase. In mice, the process of cytidine deaminase-dependent inactivation of gemcitabine was diminished by a treatment of antibiotic ciprofloxacin [171]. One study looked into the effects of bacteria on the efficacy of commonly used chemotherapies. The efficacy of 1/3rd of the drugs greatly hindered by particular bacteria, while the efficacy of six others was found to be increased by the same bacteria [172]. These data collectively indicate the potential of tissue-resident microbe in altering the efficacy of the chemotherapeutic drugs, and thereby altering the course of disease progression.

### 8.6. Other Cancer-Promoting Roles of Microbiota

Among other functions, intra-tumor bacteria can facilitate the metastasis of breast cancer cells in mice by improving cancer cells’ resistance to mechanical stress in the circulation, allowing the increased survival of circulating tumor cells [173]. Specifically, intracellular bacteria can reorganize the actin cytoskeleton in the circulating tumor cells, thereby enhancing their survival by increasing tolerance to mechanical stress in the circulation. The intra-tumor bacteria promoted metastasis but were not associated with tumor initiation. The effects of microbiota have been reported on cancer-associated genes as well. For instance, the tumor-suppressor p53, often regarded as the ‘guardian of the genome’, is influenced by the microbiota residing in the tumor microenvironment [174]. The gut microbiota suppressed the tumor-suppressive effects of p53. Specifically, microbial metabolite gallic acid alone triggered a malignant phenotype in gut-sterilized animals and p53-mutant organoid model. Since different bacterial populations colonize the GI tract in a site-specific manner, the regiospecific influence of bacterial populations thus may explain the differential effects of p53 along the length of the GI tract [174]. Finally, gut microbial influence on CRC development has been implicated through epigenetic changes by modulation of regulatory miRNA expressions, DNA methylation, histone modifications, and chromatin remodeling [175]. However, direct evidence linking microbiota-induced cancer induction through epigenetic modifications remains critically lacking.

## 9. The Microbiota, Immunity, Cancer Axis

Infection and chronic immune responses at the tumor site precede 15–20% of all malignancies [176]. The microbiota play an essential role in the immune priming of the host and have been attributed to the evolution and development of the immune system [177]. Evidently, germ-free mice have an underdeveloped immune system and altered physiological and metabolic phenotype than their normal counterparts [178]. Alterations of the gut and tissue-resident microbes have been attributed to several chronic inflammatory conditions, including cancers.

Various experimental models have been utilized to study the association between specific microbes, host immune response, and cancer. CRC-prone mice, when gavaged with *Peptostreptococcus anaerobius* demonstrate an increased risk of CRC. *P. anaerobius* interacts with TLR2 and TLR4 on the colonocytes, and modulates tumor-associated macrophages, granulocytic tumor-associated neutrophils, and myeloid-derived suppressor cells to promote CRC [6]. By exclusively binding to cancer cells using cell-surface integrins but not to normal colonocytes, *P. anaerobius* can promote NFκB-dependent inflammation and PI3K-Akt-dependent cell proliferation. The enterotoxigenic *B. fragilis* can aggravate the Th-cell immune response, and *F. nucleatum* can upregulate inflammatory and oncogenic responses to trigger CRC in mice [4]. The increased population of *Fusobacterium nucleatum*, *Peptostreptococcus anaerobius*, and *Bacteroides fragilis* has been identified as a risk factor for CRC due to the promotion of cell proliferation, DNA damage, inflammation, and protecting tumors from immune reactions [179]. Using an IL-10 knockout mouse, it was demonstrated that the Enterobacteriaceae family was upregulated >100-folds and that individual inoculation of *E. coli* and *Enterococcus faecalis* resulted in colitis in IL-10 KO mice. Furthermore, *E. coli* could induce colorectal cancer in those mice after treatment with procarcinogen azoxymethane [3]. It was also demonstrated that while IL-10 mutant mice develop spontaneous colitis under normal conditions, housing IL-10 mutant mice in a pathogen-free facility resulted in lesser disease severity [180]. Data indicate that microbiota-associated uncontrolled Th1 response is likely responsible for exacerbating colitis and adenocarcinoma formation in IL-10 mutant mice [181]. Given the immunosuppressive role of IL-10, these experiments demonstrate that a lack of immunosuppressive characteristics may trigger tumorigenesis. Another study showed that the experimental blockage of TNF-α limits the development of colitis and colorectal cancer, and the gut microbial profile of such mice was different from that of the normal counterpart [182]. However, these changes were not observed when animals were housed together, and microbiota-transplantation from anti-TNF-treated mice to normal mice showed reduced carcinogenic activity. Further, mice with transforming growth factor-β1 knockout (TGF- β1 KO) possess colorectal inflammation and cancer; however, germ-free mice with TGF- β1 KO are free from such pathologies [183]. In APC^min/+^ mice and a colitis-associated cancer mouse model, commensal *E. coli* increases the IL-17C expression, promoting tumor cell proliferation by suppressing apoptosis, inducing BCLXL, and recruiting tumor-promoting lymphocytes [184].

TLR4 plays an essential role in the mucosal inflammation associated with gut microbiota, and is a lucrative target for mitigating intestine-derived pathological insults associated with metabolic disease [185,186,187]. Mice lacking TLR-4 are substantially less likely to develop colitis-related cancer [188]. Azoxymethane treatment enhanced β-catenin activation and led to more spontaneous tumors in TLR-4 overexpressed animals relative to normal counterparts [189]. Similarly, mice deficient in MyD88, a critical TLR adapter protein, are also protected from microbiota-associated CRC formation [190]. TLR4, in association with gut microbiota, can trigger the development of HCC [191]. While antibiotic-treated mice, germ-free mice, and mice with TLR4 KO had a lower burden of HCC, wild-type mice treated with diethylnitrosamine and CCl_4_ had an increased tendency of HCC development. Increased endotoxin levels associated with gut dysbiosis and an elevated population of Gram-negative pyrogenic bacteria are risk factors for developing mucosal inflammation and subsequent carcinogenic responses. The role of dysbiosis-associated TLR4-signaling in the pathogenesis of colorectal cancer was also reported were TLR4 under the influence of protease cathepsin K, triggers M2 polarization of macrophages and promotes tumor metastasis [192].

The effects of microbiota in triggering pancreatic ductal adenocarcinoma (PDAC) by modulating innate and adaptive immune responses have been demonstrated using mice models. It was shown that the PDAC-associated microbiota is different from its healthy counterpart and that flushing gut bacteria prevent carcinogenesis and transfer of bacteria from cancer-bearing animals’ reverse tumor protection [193]. Importantly, it was revealed that flushing of gut bacteria resulted in immunological reprogramming by a decrease in myeloid-derived immunosuppressive cells and an increased M1 macrophage differentiation, which resulted in the activation of CD8^+^ T cells and Th1 differentiation of CD4+ T cells. Since PDAC-associated microbiota generated an immunotolerant microenvironment by differentially activating TLR-associated receptors in the monocytes, flushing bacteria increased immunotherapeutic efficacies against PDAC. Using genetically modified mice with lung adenocarcinoma that have point mutation of Kras and loss of p53, others have demonstrated that bacterial overgrowth and composition in the airways are related to inflammation in lung cancer [138]. In myeloid cells, the lung cancer-associated microbiota triggered the Myd88-dependent generation of IL-1β and IL-23 and induced proliferation and activation of diverse T cell populations responsible for IL-17 production, promoting neutrophil infiltration and inflammation in the lung tissue. Indeed, attenuation of tumor growth was possible in germ-free mice or when normal mice were subjected to antibiotic treatment for the depletion of the microbiota. This was supported by an earlier study showing that mice treated with aerosolized antibiotics reduced melanoma B16 lung metastases in association with a decrease in Tregs populations and an increase of T cell and NK cell activation, which necessarily renders immune protection in the tumor microenvironment [194]. The role of microbes in lung cancer-associated immunoregulation was further demonstrated when tumor regression and increased immunity were observed when bacterial isolates from the lungs of antibiotic-treated mice were inoculated in tumor-bearing mice and aerosolized probiotic strain *Lactobacillus rhamnosus* was treated to mice with tumors.

Gut microbiota could directly or indirectly affect infection-associated cancer by altering the host immune system. Such a hypothesis has been put forward based on the observations that germ-free mice show severe immunocompromised nature. Germ-free mice demonstrate a decrease in αβ T cells, CD4+ and CD8+T cells, Th1 cells, Th17 cells, CD4+CD25+ T cells, γδ T cells, and antibody-secreting plasma cells in various parts of the body including intestine, spleen, lymph nodes and in the circulation when compared to specific pathogen-free mice, and demonstrate reduced expression of Angiogenin-4, regIIIγ, secretory IgA, TLR9, MHC class II, and IL25 at the intestinal cells [63,195], resulting in poor anti-cancer protection by the immune system.

One of the emerging arenas of microbe-associated cancer is related to inflammasomes which remains controversial as either promoter or suppressor of cancers. The precise consequence of inflammasome activation is dictated by several factors, including the inflammasome’s expression pattern, effector molecules of the immune system, tumor microenvironment, and specific tumor type and stage [164]. Inflammasome protein ablation, such as NLRP6, results in the overgrowth of colitogenic microbes that trigger colon inflammation and advanced CRC development by upregulating chemoattractant CCL-5 from epithelial cells in an influx of inflammatory IL-6-producing immune cells and increased epithelial proliferation [196]. The inhibition of IL-6 signaling reduces inflammation and tumor burden, while also preventing the overgrowth of colitogenic bacteria.

The inoculation of enterotoxigenic *Bacteroides fragilis* causes an IL-17-dependent increases in carcinogenesis in the distal colon in CRC susceptible mice [7]. Mice with enterotoxigenic *B. fragilis* inoculation also develop a proinflammatory milieu marked by STAT3 activation, IL-17-dependent NF-κB activation, enhanced WNT-catenin signaling, E-cadherin cleavage, and proliferation epithelial cell proliferation [197]. Indeed, others have demonstrated that in HT29/c1 and T84 colonic epithelial cells, the pure *B. fragilis* toxin (BFT) upregulates spermine oxidase, producing ROS and DNA damage [198]. Since the polyamine catabolic enzyme spermine oxidase is strongly inducible by inflammatory stimuli, conventional mice with *Bacteroides fragilis*-induced inflammation showed a reduction in mucosal injury and cell proliferation when treated with a pharmacological inhibitor of polyamine catabolism.

Due to gut barrier dysfunction, the commensal microbiota can promote higher IL-23 and IL-17, IL-22, and IL-6 expression in colon adenoma animal models, and antibiotic therapy or genetic deletion of IL-23 abrogates carcinogenesis [199]. Using transgenic mice susceptible to polyp formation, it was shown that gut barrier defects could facilitate commensals to trigger inflammatory neutrophil accumulation that supports cancer growth [200]. The abolition of gut microbes by antibiotic treatment can reverse polyp formation, whereas reintroducing gut microbiome from polyp-bearing animals can trigger polyp development.

Despite these pieces of evidence, gut microbes’ role in cancer initiation and promotion remains underexplored and may depend on the experimental design. For instance, although the majority of the studies infer a direct causative role of microbiota with cancer initiation, an earlier study indicates the role of gut microbes only in caner promotion through exclusively inflammation-associated mechanisms [201]. Using azoxymethane and dextran sulfate sodium treated mice, it was shown that germ-free mice had hyper cell proliferation and no visible epithelial layer repair, but specific pathogen-free animals are protected from mucosal damage after one month of treatment. In these germ-free animals, KO of TLR/MyD88 alleviates colitis and slows tumor development, indicating that the TLR/MyD88 system is associated with both microbe-dependent and independent processes in inducing inflammation-associated tumorigenesis. Although these studies do not provide any complete mechanistic insight regarding how opportunistic infections by tissue-resident microbes promote tumorigenesis by influencing the immune response but provide an understanding of how altered immune response under the influence of microbes can foster an environment that favors cancer.

## 10. Pathoadaptive Mutations and Improved Colonization Efficiency of Microbiota

The integral role of gut microbiota in human health and disease has been recognized, but the microbial pattern associated with eubiosis and dysbiosis remains unknown. This is primarily since the good, bad, and ugly aspects of microbes are condition specific. Indeed, bloom of bacteria, otherwise considered commensal, are observed associated with cancers. For instance, gut commensals like *A. muciniphila*, which is generally considered beneficial and a next-generation probiotic, are highly abundant in cancer patients [202,203,204,205]. Therefore, it is likely that tissue commensals, under specific suitable microenvironment, can cause opportunistic infections leading to chronic diseases. Apart from evading host immune surveillance, two of the fundamental reason which can turn gut commensals to facilitate opportunistic infections are specific pathoadaptive mutations and improved colonization potentials. Aggravated tissue-specific microbial populations can promote long-term host inflammatory and oxidative responses, potentially underlying cancer progression. Increased mutation rates influenced by adaptation pressure, the strength of mutator alleles, self and interacting bacterial population size and competition, migration, and the heterogeneity of spatiotemporal microenvironment have all been linked to how commensal symbionts can gain an opportunistic phenotype under altered physiological conditions and promote host-tissue opposing phenotype. Pathoadaptive mutations are generally related to activities that regulate adaptation to its microenvironment or alteration, availability of preferred nutrients, immune evasion fitness, or efficient colonization via biofilm formation.

In *E. coli*, mutations in nutrient utilizing genes have been linked to increased colonization and adverse effects on the host [206]. Gain of function mutation in the histone protein HUα^E38K, V42L^ in commensal *E. coli* K-12 promotes a pathoadaptive transformation that promotes biofilm formation by changes in transcription profile [207]. These mutations can promote otherwise commensal *E. coli* K-12 to cause disruption of phagosome as a survival strategy and increase bacterial invasive behavior. Random mutations fundamental to evolution and adaptation can activate dormant genes, which confers better colonization potentials. Activation of *blg* operon that codes for functions related to β-galactosidase uptake and utilization has been reported in *E. coli* due to random mutation [208]. Increased virulence of gut commensals can also be achieved by acquired plasmid and remain independent of the cellular genome. Genomic comparison of non-pathogenic commensal *E. coli* strain with enterotoxigenic *E. coli* revealed plasmid coding for virulence factors like toxin production, a unique fimbrial system for mucosal attachment, and a novel type I secretion system for maintaining fimbriae organization which collectively increase pathogenic potentials of *E. coli* [209]. An acquired plasmid can provide pathoadaptive benefit to bacteria by improving virulence, improved niche-specific fitness, and better metabolism of available nutrients, collectively conferring the ability of stable colonization [210].

Data from the genomes of 1163 *Staphylococcus aureus* isolate from 105 infected individuals’ nasal swabs indicate that commensal bacterial infections can emerge due to mutations that boost commensal bacteria’s spontaneous adaptive evolution [211]. These mutations were mainly associated with the regulation of surface antigen, toxin production, quorum signaling, abscess formation, and host-derived antimicrobial peptide. Different strains of zoonotic pathogen *Streptococcus suis* isolated from patient samples demonstrate increased mutation rates relative to isolates from closely-related carriers [212]. In pathogenic isolates with A/T-rich small genome sizes, a stronger bias towards G/C to A/T changes was observed. Select mutations can confer gain of pathogenic traits within members of commensal populations. For instance, earlier studies demonstrate that rare pathoadaptive mutations in the otherwise benign *H. influenzae* population in the nasopharynx can cause meningitis [213]. Another study used infectious methicillin-resistant biofilm-forming *S. epidermidis* and its non-infectious non-biofilm forming counterpart to demonstrate single nucleotide polymorphism in the orthologous genes associated with resistance to host oxidative injury [214]. These mutations protect the bacteria from the host immune response and confer properties for improved colonization. Random repetitive mutations can also facilitate better adherence of the bacteria to the host tissue. In one study, *E. coli* and *B. subtilis* were positively and negatively selected for binding to pancreatic cancer cells or normal pancreatic cells after induction of random mutations [215]. Mutant *E. coli* and *B. subtilis* were bound to cancer cells 10–25 times more than normal cells. At the same time, mutations in genes related to biofilm formation and type I pilus were identified, which likely facilitated improved adhesion of the bacteria to the cancer cells. In gastric cancer-causing *H. pylori*, extensive mutations in several genes have been identified, improving colonization efficiency and drug resistance [216].

Gut commensals protect from invading opportunistic infection-causing bacteria by resisting their colonization. Long-term chemotherapy can deplete beneficial gut commensals and promote colonization of pathogenic spp. For instance, in patients with acute myeloid leukemia undergoing chemotherapy and antibiotic treatment, due to depleted anaerobic bacterial populations and a rise in potentially harmful aerobic *Enterococci*, the patients cannot maintain colonization resistance [217]. The causal relationship between improved colonization of tissue-resident microbes with various types of cancer has been discussed above. Although the direct link between such microbes with cancer initiation and progression remains underexplored, their high nutritional fitness can explain improved colonization efficiency. Efficient utilization of the available nutrient has been proposed as an adaptive strategy that confers better bacterial colonization. In the ‘nutrient-niche hypothesis’, it is suggested that a given microorganism will fit into a complex community and will be able to overgrow only if it is capable of using at least one limiting resource better than other members of the same community, assuming that all other nutrients are equally available to all members [218]. Another hypothesis, known as the ‘restaurant hypothesis’, claims that certain microorganisms can profit from the metabolic processes of other microbes in the same population.

Preferred nutrient availability is a critical limiting factor for microbial colonization fitness and overgrowth. Using streptomycin-treated mice, it was demonstrated that based on nutrient intake, *E. coli* could limit the colonization of foreign invading *E. coli* [219]. Pre-colonization of mice with diverse sugar-utilizing probiotic *E. coli* HS and *E. coli* Nissle 1917 can prevent subsequent colonization by the enteropathogenic *E. coli* EDL933 through mechanisms involving better nutrient utilization. Increased Firmicutes-to-Bacteroidetes ratio is commonly associated with various types of cancers [220]. Colonization experiments on germ-free mice were performed using *B. thetaiotaomicron* (Bacteroides) and *Eubacterium rectale* (Firmicutes) to understand the nutritional competition between two predominant phyla [221]. In the presence of *B. thetaiotaomicron*, improved metabolic flexibility was shown by *E. rectale* through the downregulation of glycan-metabolizing genes and upregulating glycolysis; whereas, in the presence of *E. rectale*, *B. thetaiotaomicron* adapted to use a wide variety of sugars by upregulating genes encoding glycoside hydrolase enzymes and also induced increased mucosal glycan production that only *B. thetaiotaomicron* can utilize.

Cross-signaling through bacterial metabolites plays a vital role in improved colonization. For instance, bacteriocin from *Listeria monocytogenes* inhibits the growth of commensal *Prevotella copri*, which is known to utilize intestinal mucus as a nutrient source [222]. Loss of *P. copri* results in a thicker mucus layer which protects the pathogenic *L. monocytogenes* from the mucosal immune response. Similarly, bacterial cross metabolic signaling was reported in a mice model where polysaccharide A secreted from commensal *Bacteroides fragilis* protects mice from *Helicobacter hepaticus*-induced experimental colitis [223]. Mice colonized with *B. fragilis* that is unable to produce polysaccharide A had increased mucosal inflammation, which could be suppressed by the experimental treatment with *polysaccharide A*. Analysis of bacterial metabolite patterns (e.g., trimethylamine, secondary bile acids, hydrogen sulfide) of ~3000 metagenomic and metatranscriptomic data reveals that the pathofunctions of autochthonous microorganisms can cause chronic diseases when invading pathogens are absent [224]. In fact, commensals carrying genes related to various pathofunctions are upregulated in patients with CRC, liver cirrhosis and IBD.

Collectively, under disease conditions, the microbial population diversity and abundance can be influenced by the altered tissue microenvironment. Contrarily, altered tissue metabolic homeostasis can promote increased colonization of pathobionts which can, in turn, promote cancer development by inducing localized inflammatory and oxidative injury.

## 11. Microbiota-Centric Strategies against Cancer

Due to gut microbes’ important role in maintaining human health and the pathogenesis of chronic diseases, alteration of microbiota by strategies including using diet, prebiotics, probiotics, antibiotics, and fecal microbiota transplantation (FMT) has emerged as a lucrative prophylactic and therapeutic strategy against cancers. Nevertheless, since longitudinal clinical studies comprising of the large case-control cohort to evaluate the therapeutic and safety aspect of microbiota-centered anti-cancer strategies are critically lacking, and since, long-term probiotic/synbiotic administration could be associated with increased risk of adverse GI symptoms [225], utmost care should be taken for the prolonged therapeutic use of microbes against chronic diseases. Collectively, the microbiota-centric anti-cancer strategies can be broadly categorized into the following:

### 11.1. Dietary Strategies

Diet is the most critical factor that regulates the gut microbiota. A plethora of studies indicates the health-promoting or deteriorating role of dietary quality and quantity due to effects on gut microbiota. In contrast to calorie-rich Western diets, the Mediterranean diet, mainly containing fruits and vegetables, whole grains, herbs, spices, seafood, and less consumption of red meat, has been shown to improve gut microbial diversity. Especially lower populations of Firmicutes, Proteobacteria, increased *Bifidobacterium*, *Lactobacillus*, *Akkermansia*, *Fecalibacterium*, lower mucosal inflammation, and gut barrier dysfunction have been observed due to prolonged consumption of the Mediterranean diet [226]. Using female monkeys, it was demonstrated that >31-mo supplementation of Mediterranean diet results in a significant increase in *Lactobacillus* and a decrease in reactive oxygen species metabolites in the mammary gland relative to monkeys consuming a Western diet [227]. Data from the large-scale European Prospective Investigation into Cancer and nutrition (EPIC) study comprising 5,296,617 person-years of follow-up suggest that adherence to the Mediterranean diet is associated with a 33% reduced risk of gastric cancer [228]. Similar observations from the MOLI-SANI study, including 24,325 subjects consuming a Mediterranean diet, reported a reduced level of inflammatory markers in the circulation, a risk factor for cancers [229]. In line with pre-clinical studies indicating the direct cancer risk with prolonged consumption of a calorie-rich diet and subsequent alterations in gut microbiota, several clinical studies have focused on calorie restriction (CR) to reduce cancer risk. Using obese patients who have a laparoscopic sleeve gastrectomy, one study demonstrated that supplementation of a very low-calorie diet followed by a gradual transition to a high-fiber, low-fat, and CR diet results in malabsorption of nutrients in the intestine [230]. These effects were associated with an increased abundance of butyrate-generating firmicutes such as *Butyrivibrio fibrisolvens*, *Clostridium saccharolyticum*, *Eubacterium limosum*, and *Blautia hydrogenotrophica*. Another study treated glioblastoma patients with 72-h water-only fasting followed by a 21-day ketogenic diet enriched with vitamins and minerals [231]. Apart from tumor regression and decreased tumor cell invasion, the glycemic index, urine ketone levels, serum insulin, and triglyceride levels were normalized, likely due to favorable modulation of the gut microbes.

The consumption of polyphenol-rich food has been implicated in reducing cancer risks by mechanisms associated with improvements in gut microbiota. We have demonstrated that catechin-rich green tea can limit the clinical progression from nonalcoholic steatohepatitis to HCC by mitigating hepatic inflammation and pro-carcinogenic responses [232], in association with mitigating GI and hepatic inflammation, gut barrier dysfunction, and favorable modulation of gut microbial population, diversity, and metabolic functions [185,186,187]. The mechanism through which a polyphenolic-rich diet can be preventive of carcinogenesis through the favorable modulation of microbiota can include the modulation of hormonal regulation, limiting oxidative and inflammatory injury, and maintaining cellular proliferative/apoptotic homeostasis [233]. Although a detailed discussion on the gut microbiota-associated anti-cancer effects of polyphenols is out of the scope of the manuscript, a detailed discussion on the impact of polyphenols on gut microbiota has been reviewed elsewhere [234,235,236,237].

### 11.2. Prebiotics

Although prebiotics falls under the diet category, their distinct role in influencing the growth of gut commensals and probiotic bacterial spp. makes them a suitable prophylactic approach against chronic disease. In general, their beneficial role in limiting carcinogenesis includes stimulating the growth of beneficial bacteria, production of SCFA, affecting gene expression patterns opposing proliferation and tumorigenesis, enhancing nutrient absorption, modulating xenobiotic metabolism, lowering oxidative and inflammatory injury, and building robust immune response [238]. Specific prebiotic constituents of whole foods generally include galactooligosaccharides, fructooligosaccharides, oligofructose, inulin, and dietary fibers. SCFA is generated from the anaerobic fermentation of dietary fibers by gut microbes (e.g., Faecalibacterium, Clostridium, Ruminococcaceae, Eubacterium, Roseburia) [234]. Although the range of anti-cancer activities of different SCFA (e.g., butyrate, propionate, acetate) may differ, they are commonly associated with energy supply to the colonocytes, histone modification resulting in enhancement of transcriptional activities, suppression of cell proliferation, enhancement of apoptosis, elimination of damaged cell, inhibition of growth factor secretion, and lowering inflammation and oxidative injury [239]. Butyrate derived from dietary fibers can ameliorate colorectal cancer by limiting cancer-associated gut microbiota through anti-inflammatory response by suppressing histone deacetylase in mice [240]. Although detailed discussion on the anti-cancer mechanisms is out of the scope of the chapter, they broadly involve the inhibition of cellular proliferation, migration, invasion, migration, angiogenesis, and survival by inhibition of histone deacetylase; promoting apoptosis by triggering mitochondrial caspase pathway or by inducing cell death through cell-surface receptors (e.g., Fas, TRAIL); CDK-depended cell cycle arrest by modulation of miRNA responses; inhibition of specific oncogenic responses (e.g., HEY1); and inhibition of transcription factors associated with pro-inflammatory responses (e.g., NFκB, STAT3) [241]. Using germ-free mice, one study demonstrated that dietary fiber protects against CRC in a microbiota- and butyrate-dependent mechanisms [242]. Specifically, germ-free mice were colonized with wild-type and mutant butyrate-producing *Butyrivibrio fibrisolvens* and then supplemented with dietary fiber. The resultant butyrate produced from the fibers was reported to accumulate and inhibit histone deacetylase rather than being metabolized by the tumor cells, which caused increased cell apoptosis and less cell proliferation.

Large-scale clinical trials on the beneficial effects of prebiotics on cancer risk or progression are lacking. However, one systematic review and meta-analysis of 21 case-controlled studies comprising more than 2 million subjects indicated that higher consumption of dietary fiber is associated with reduced breast cancer risk [243]. Specifically, the data suggested that the risk of breast cancer was inversely associated with soluble fiber consumption, and both premenopausal and postmenopausal breast cancer risk was inversely related to increased total fiber intake. In support of the beneficial role of dietary fibers against cancers, appropriate studies have been summarized in Table 2.

### 11.3. Probiotics

Probiotics are ‘beneficial’ live bacteria commonly belonging to the genera *Lactobacillus* and *Bifidobacterium* and yeasts such as *Saccharomyces boulardii*. The beneficial effects of probiotic supplements in cancer are mostly prophylactic, including preventing biotransformation of inactivated pre-carcinogens to reactive carcinogenic species, lowering liminal pH by SCFA production, limiting mucosal inflammation and enhancing immune function, and inhibiting mutagenesis by degradation and/or deactivation of reactive species [238]. Mice and human colon carcinoma cells infected with live *Lactobacillus casei* resulted in a significant reduction in cell proliferation in a time and concentration-dependent manner [253]. *L. casei* induces apoptosis by upregulation of TNF-related apoptosis-inducing ligand and reducing the expression of anti-apoptotic protein survivin. Another widely used probiotic bacterium, *Bifidobacterium*, can also be helpful against cancer. Data show that upon flushing intestinal bacteria by antibiotic treatment, tumor-bearing mice that generally respond to anti-CD47 therapy do not show any response [254]. However, those mice, upon supplementation of a probiotic cocktail containing *Bifidobacterium* species consisting of *B. bifidum*, *B. longum*, *B. animalis* subsp. *lactis*, and *B. breve*, respond to the anti-cancer treatment. Indeed, patients with non-small-cell lung cancer show an abundance of *Bifidobacterium bifidum* in the gut, who respond to immune-checkpoint inhibitor anti-cancer therapies [255]. In mice, it has been further demonstrated that only specific strains of *B. bifidum* can reduce tumor burden synergistically with PD-1 or oxaliplatin therapies. In 85 patients with locally advanced nasopharyngeal cancer, supplementation of a probiotic consortium containing *Bifidobacterium animalis*, *Lactobacillus plantarum*, *Lactobacillus rhamnosus*, and *Lactobacillus acidophilus* demonstrated a significant reduction of oral mucositis and improved immune response [256]. Supplementation of VSL#3, a commercial probiotic formulation composed of *Lactobacillus acidophilus*, *Lactobacillus plantarum*, *Lactobacillus casei*, *Lactobacillus delbrueckii* subspecies *bulgaricus*, *Bifidobacterium breve*, *Bifidobacterium longum*, *Bifidobacterium infantis*, and *Streptococcus salivarius* subspecies *thermophilus*, has been reported to attenuate several chronic GI conditions [257]. Despite its efficacy in restoring healthy commensal microbial populations and reducing colitis-associated inflammation, experimental data using colitis susceptible IL-10^−/−^ mice have shown that VSL#3 may increase tumor multiplicity, penetrance, histopathological dysplasia, and adenocarcinoma invasion under azoxymethane-treatment [258]. Nevertheless, others have reported that supplementation of VSL3# with anti-inflammatory drug balsalazide in mice with colorectal cancer induced by azoxymethane/dextran sodium sulfate reduces tumor burden in association with increasing Bax/Bcl-2 ratio and lowers IL6/STAT-3 signaling [259].

Due to the intestinal-level benefits of probiotic strains, efforts have been made to develop functionally enhanced synthetic probiotics in recent years. For instance, using the lactic acid bacterium *Pediococcus pentosaceus*, researchers developed an efficient delivery method for CRC treatment. The modified *P. pentosaceus* was equipped with twin gene cassettes encoding protein P8 linked to a secretion signal peptide and a complementation system [260]. P8 is a stress-induced protein known to possess anti-proliferative activities [261]. Supplementation of the modified *P. pentosaceus* in mice with CRC significantly reduced tumor lesions associated with improving the abundance of gut commensals.

The mechanisms through which probiotics likely exert anti-cancer effects are not well defined but are primarily attributed to the restoration of commensal microbial population and a favorable antitumor immune response at the intestinal mucosa. Evidence suggests that specific molecules derived from gut commensals can influence tumor promotion or inhibition. Indeed, a recent study has shown that siderophore ferrichrome isolated from *Lactobacillus casei* possesses superior anti-cancer effects than cisplatin and 5-fluorouracil. The mechanisms involve the induction of apoptosis by activating c-jun N-terminal kinase [262]. *Bacillus subtilis*-derived competence and sporulation factor, which aids in bacterial quorum-sensing, can activate p38 MAP kinase and protein kinase B mediated survival signaling and trigger the expression of cytoprotective heat shock proteins that limit oxidative stress injury and barrier dysfunction at the intestinal epithelia [263]. Others have reported that two novel proteins (75 and 40 kDa) isolated from *Lactobacillus rhamnosus* GG can activate protein kinase B, suppress cytokine-induced apoptosis, and inflammatory injury to intestinal epithelial cells [264]. Polyphosphate isolated from *Lactobacillus brevis* reduced gut barrier dysfunction, degradation of F-actin and E-cadherin induced by oxidative damage in a mechanism involving P38 MAPK activation, and protects mice from NFκB-dependent inflammatory injury caused by dextran sodium sulfate [265]. In another study, cell-free supernatant of three different strains of *Lactobacillus rhamnosus* isolated from breast milk demonstrated potent antioxidant activities, down-regulated the expression of Bcl-2, whereas up-regulated BAD, BAX, caspase3, caspase8, caspase9 in HeLa cells [266]. These reports collectively demonstrate that the anti-cancer properties of probiotic bacteria may not be associated with the functional attributes of the live bacteria, instead could as well be attributed to the bacterial metabolites.

Since metabolic endotoxemia is a risk factor for developing hepatocellular carcinoma, microbial interventions that improve gut barrier functions and limit endotoxin translocation along the gut-liver axis could be a prophylactic strategy against HCC. In 30 cirrhotic patients with hepatic encephalopathy, 8-wk supplementation of *Lactobacillus* GG reduced metabolic endotoxemia reduced the level of serum TNFα while reducing the populations of *Enterobacteriaceae*, which is attributed to chronic liver diseases [267]. Finally, a meta-analysis of 7 clinical studies indicated that probiotic supplementation could potentially be a preventive strategy against surgical infections in patients with colorectal cancer [268]. Other experimental evidences of probiotics supplementation as an effective anti-cancer strategy have been provided in Table 3.

### 11.4. Synbiotics

Since probiotics and prebiotics have health beneficial effects, synbiotics, i.e., the combined formulations of probiotics and prebiotics, are also expected to have superior prophylactic and therapeutic effects against chronic diseases like cancers. However, there is a critical lack of direct experimental evidence of synbiotic formulations limiting cancer risk. Most such studies have focused on animal CCR models induced by azoxymethane treatment. Azoxymethane is metabolized by cytochrome P2E1 to methylazoxymethanol, which causes DNA mutations and negatively alters the signaling pathways related to carcinogeneses like K-ras, Src/PI3K/Akt, beta-catenin, TGFbeta, and p53 [279]. One of the earliest studies used a combination of 5% (*w/w* in diet) inulin and B. longum (4 × 10^8^ cells/g diet) on mice having CCR induced by colon carcinogen azoxymethane [280]. The combined therapy was better efficacious in limiting the numbers of colonic aberrant crypt foci compared to individual treatments. Another study used a symbiotic formulation of *L. acidophilus* and *B. lactis* (1 × 10^10^ cfu/g) and resistant starch (10% *w/w* in diet) in male azoxymethane-treated Sprague-Dawley rats with CRC [281]. Supplementation of the formulation resulted in increased cell apoptosis, improved mucosal histopathological features, increased fecal population of *Bifidobacterium* and *Lactobacillus*, collectively increasing the capacity to limit carcinogenic insults. To better understand the effects of symbiotic formulations on CRC immune response, one study tested a symbiotic formulation of L. rhamnosus, B. longum, and inulin-based oligofructose in a mice CRC model [282]. The symbiotic formulation reduced the incidence of primary tumors in association with suppressed cytotoxicity induced by Natural Killer cells, elevated immunosuppressive phenotype by increased secretion of IL-10, and decreased IFN- γ and lymphocyte proliferation.

The beneficial effects of symbiotic formulations have also been demonstrated in humans. A randomized, double-blind, placebo-controlled trial used a symbiotic combination of oligofructose-enriched inulin, *L. rhamnosus*, and *B. lactis* to test whether the formulation can reduce CRC risk. In study subjects comprising CRC patients and polypectomized individuals, 12-wk supplementation of the symbiotic formulation reduced proliferation of the colonocytes, improved gut barrier function, reduced IL2 levels, and elevated the production of IFN-γ. Subjects with the synbiotic treatment also had significantly increased *Bifidobacterium* and *Lactobacillus* fecal populations and decreased abundance of *Clostridium perfringensi* [283]. Beyond limiting cancer risk, one study demonstrated that synbiotics could reduce chemotherapy-associated adverse effects in 30 esophageal cancer patients undergoing chemotherapy by favorably altering the gut microbiota [284]. A synbiotic formulation consisting of *B. breve* (10^8^ cells), *L. casei* (10^8^ cells), and galacto-oligosaccharides (15 g/day) reduce the abundance of *C. difficile*, *Enterococcus*, *Staphylococcus*, and methicillin-sensitive coagulase-negative *Staphylococcus aureus* while elevating the abundance of *Bifidobacterium* and *Lactobacillus*. Patients receiving the synbiotic formulation had lower frequencies of lymphopenia, febrile neutropenia, and reduced incidence of diarrhea in association with increased fecal concentrations of SCFA. These evidences collectively indicate exciting opportunities for combined probiotic and prebiotic strategy in cancer.

### 11.5. Antibiotics

Antibiotics disrupt and deplete gut microbiota. In general, higher early childhood antibiotic treatment makes an individual prone to obesity at a later age [1]. However, cancers arising from microbial infections or gut microbial imbalance can be potentially treated with specific antibiotic treatments. A recent study demonstrated how a 7-d antibiotic treatment affects the gut microbiota of healthy subjects for up to 31 months. For 1-wk, 13 men were given no therapy or an antibiotic cocktail of ciprofloxacin, vancomycin, and metronidazole [285]. The broad-spectrum antibiotics had a significant short-term influence on the gut microbiota, resulting in a loss of diversity and substantial changes in microbial community composition. Antibiotics also lowered the abundance of bacterial taxa that perform vital metabolic tasks (e.g., butyrate production). After 8-31 months, the microbiota demonstrated a spectacular return to baseline. Still, community composition was frequently altered from its initial condition, indicating that antibiotics have substantial and long-lasting effects on gut microbiota.

The cancer-associated antibiotics can be broadly divided into four classes depending on their mode of function, viz., inhibition of bacterial cell-wall formation (e.g., cephalosporins), inhibition of bacterial replication and transcription (e.g., dichloroacridine, quinolones), inhibition of bacterial translations (e.g., tetracyclines), and cell-wall pore-forming antibiotics (e.g., Aminoglycosides) [286]. Cancer-associated antibiotics can play a dual role by depleting cancer-associated microbiota and directly inhibiting host cellular processes related to cancer formation. The polyether ionophoric antibiotic salinomycin can hinder the growth of Gram-positive bacteria, including the notorious methicillin-resistant *Staphylococcus aureus* and methicillin-resistant Staphylococcus epidermidis. Experiments using CRC-related primary tumor-initiating cells show that salinomycin can exert better anti-proliferative effects than 5-fluorouracil and oxaliplatin chemotherapy [287]. Salinomycin can induce apoptosis in CRC cells in a patient-derived mouse xenograft model by mitochondrial dysfunction and generation of reactive oxygen species. A few clinical case studies have also proven the usefulness of salinomycin in therapy-resistant cancer patients; for example, a patient with metastatic invasive ductal breast cancer treated with salinomycin experienced clinical tumor regression [288]. Interestingly, another set of data from broilers shows that salinomycin, apart from increasing the SCFA levels in the cecum, also depletes the abundance of lactic acid bacteria, coliform bacteria, *Clostridium perfringens*, and *Lactobacillus salivarius* in the cecum while improving the abundance of lactose-negative *Enterobacteria* in the ileum [289]. The broad-spectrum fluoroquinolone-class of antibiotic Gemifloxacin suppressed motility and invasion of human breast adenocarcinoma cells and induced mesenchymal to epithelial transformation as an effective anti-metastatic process [290]. GMF also inhibited the activation of NFκB, and tumor necrosis factor (TNF-α)-induced cell migration and invasion. The effects of gemifloxacin on gut microbiota were studied in 10 healthy volunteers who received 320 mg gemifloxacin for seven days, whereas five others received a placebo [291]. During gemifloxacin treatment, the abundance of Enterobacteria was reduced, as were the quantities of Enterococci and Streptococci as part of the aerobic microbiota. In contrast, only cocci and *Lactobacilli* were reduced as part of the anaerobic microflora. The microbiota returned to normal 49 days after the gemifloxacin treatment was stopped. Several other antibiotics (e.g., doxorubicin, mitomycin) that have selective microbial-depleting capacities can exert anti-cancer effects by diverse mechanisms involving inhibition of cell proliferation by inhibiting topoisomerases and regulation of cell-cycle, induction of apoptosis by mitochondrial production of intracellular oxidative stress, and activation of caspase signaling, inhibition of inflammation and cell migration [286].

Finally, chemotherapeutic agents and antibiotics are often metabolized by specific gut microbes prior to site-specific therapeutic activities. Thus, antibiotic treatment could also have adverse effects on cancer patients receiving chemotherapy. One study comprising 196 cancer patients receiving immune checkpoint inhibitor therapy reported reduced response to chemotherapy upon antibiotic treatment [292]. These findings collectively indicated that the timing of antibiotic exposure to the patients is critical in determining how the immunotherapy is affected. Broad-spectrum antibiotic treatment can potentially disturb the gut environment for a long time and reduce the efficiency of the cytotoxic T-cell response against cancer, bolstering the plausibility of antibiotic treatments’ negative impact on immunotherapy outcomes.

### 11.6. Fecal Microbiota Transplantation

In general, fecal microbiota transplantation (FMT) is oral or colonic delivery of stool samples from a healthy donor(s) to the patients for the remission of specific diseases through the replacement of unhealthy or disease-causing microbiota. Since the first experimental use to treat pseudomembranous colitis patients [293], successful therapeutic strategies with FMT have been demonstrated against different diseases, including cancers. The mechanism through which FMT could potentially be beneficial to cancer patients includes restoration of the healthy gut bacterial population (e.g., gut commensals), improvements in bacterial diversity, removal of infectious and mutagenic bacteria (e.g., *H. pylori*, *C. difficile*), modifications of gut barrier function to limit gut-to-systemic translocation of bacterial metabolites (e.g., LPS), and favorable immunological response supporting the suppression of carcinogenic processes [294].

Several studies using rodent models have demonstrated various benefits associated with FMT. Especially in mice with cancer, FMT has been shown by several studies to provide relief from cancer treatment-related distresses. For example, the changes in gut microbiota have been attributed to the colitis-associated cancers (CAC), which is the most harmful outcome of inflammatory bowel disease. In a mice model of azoxymethane–dextran sodium sulfate-induced CAC, FMT successfully restored healthy gut microbial populations and improved their diversity [295]. FMT suppressed intestinal NFκB-dependent inflammatory signaling, increased the populations of Treg cells, and attenuated the proliferation of cells in the colon. One study demonstrated that altered gut microbiota induced by antibiotic treatment or chemotherapy could be restored by FMT [296]. In C57BL6/J mice, a 7-d treatment of ampicillin alone or in combination with 5-fluorouracil I.P. injection lowered microbial diversity and richness. *Clostridium scindens* and *Faecalibacterium prausnitzii* were lowered, while the population of pathogenic *E. coli* was increased. FMT of feces from untreated healthy mice for 3-d resulted in reversal of microbiota caused by antibiotics and chemotherapy. Inline, a recent study has shown that 5-fluorouracil/oxaliplatin-mediated toxicity and TLR-dependent inflammatory injury to the intestine in mice with CT26 colorectal adenocarcinoma implantation can be mitigated by FMT from feces from healthy mice [297]. Exposure to radiation as part of anti-cancer therapy is associated with bone marrow and GI tract toxicity, giving rise to radiation syndrome. Using cancer-bearing mice undergoing radiation therapy, it was demonstrated that FMT from a healthy cohort resulted in a better survival rate of patients, increased peripheral white blood cell count, and improved GI integrity [298].

A plethora of clinical approaches has been directed towards improving patients’ responses to anti-cancer therapies using FMT. Recent studies demonstrate that FMT can confer clinical benefits in patients with metastatic breast cancer resistant to immune checkpoint blockade therapies [8,9]. In one study comprising ten patients with anti-PD-1-refractory metastatic melanoma, FMT resulted in favorable immune phenotype and gene expression profiles at the intestinal lamina propria and the tumor microenvironment [8]. FMT also improved the abundance of bacterial populations belonging to the immunotherapy-favorable *Veillonellaceae* family and lower abundance of *Bifidobacterium bifidum*, which is known to trigger T-reg cell-mediated immune tolerance. In another study intending to investigate whether resistance to anti-PD-1 therapy could be overcome by altering the gut microbiota, 15 advanced melanoma patients underwent FMT [9]. Post FMT in 6 patients, a favorable immune response was observed, including higher activation of CD8^+^ T cells and lower frequency of myeloid cells with IL-8. Additionally, restoration of healthy microbiota was observed, including an increased abundance of bacteria associated with response to anti-PD-1 therapy. Several pre-clinical and clinical studies have recently indicated the association of gut microbiota in the efficacy of anti-cancer treatments related to pancreatic ductal adenocarcinoma, hematologic cancers, metastatic melanoma, non-small cell lung cancer, and renal cell carcinoma [299]. Apart from improving the efficacy of anti-cancer therapies, FMT can also be helpful in mitigating side effects associated with anti-cancer therapies. For instance, diarrhea is a common side-effect of chemotherapeutics, and gut microbiota has been attributed to the process. In tyrosine kinase inhibitor-induced diarrhea-prone patients with metastatic renal cell carcinoma, FMT successfully resolved diarrhea in 4-wk [300]. It was further reported that the donor FMT was more efficacious than the placebo FMT for treating diarrhea, with successful microbial engraftment in subjects receiving donor feces. No side effects were observed associated with the FMT.

Multidrug-resistant (MDR) bacterial infections in cancer patients can exacerbate the rate of mortality and morbidity. Although limited in number but application of FMT in cancer patients with MDR has demonstrated varying degrees of success. A systematic review comprising 20 FMT reports from 121 patients has reported 70.3% eradication of MDR bacteria [301]. More specifically, 68.2% reduction of Gram-positive and 70.6% reduction in Gram-negative bacteria were reported. Another systematic review comprising 151 patients with MDR bacterial infection from 20 studies has reported up to 87% success rate of FMT in eradicating MDR bacterial infections [302]. In a prospective, single-center trial comprising 20 patients with various hematological diseases (e.g., acute myeloblastic leukemia, diffuse large B-cell lymphoma), FMT inhibited the intestinal growth of several antibiotic-resistant bacteria, including vancomycin-resistant *Enterococci*, carbapenem-resistant *Pseudomonas aeruginosa*, *Enterobacter cloacae*, and *Klebsiella pneumoniae*, and metallo-β-lactamase containing *Pseudomonas aeruginosa* in 75% of all the patients [303]. In another study comprising ten patients with hematologic malignancies having infection of vancomycin-resistant enterococci or carbapenemase-producing bacteria, 70% successful decolonization of MDR bacteria was achieved due to FMT [304]. Currently, 26 trials have been registered, out of which 15 are currently recruiting patients to test both safety and the efficacy of FMT in cancer (www.clinicaltrials.gov; accessed on 5 March 2022).

## 12. Conclusions

Despite that fact that the International Cancer Microbiome Consortium has indicated ‘*no direct evidence that the human commensal microbiome is a key determinant in the aetiopathogenesis of cancer*’ [305], distinct shifts in specific gut microbial populations are evident in cancer patients. Although an urgent need for large-scale longitudinal cohort studies exists to determine the role of tissue-specific microbes in cancer, microbial profiling in relation to cancer or other chronic diseases may not be a practical approach since the gut microbial population is dependent on several factors like diet, overall health condition, hygiene, lifestyle, etc. Moreover, the tissue-specific microbial population could also be disease stage-specific. For instance, distinct shifts in microbial populations have been observed in the case of a large cohort of colorectal cancer patients [4]. Out of two different profiles, intramucosal carcinoma to more advanced stages were associated with an increased abundance of *Fusobacterium nucleatum* spp., whereas multiple polypoid adenomas and/or intramucosal carcinomas were associated with increased populations of *Atopobium parvulum* and *Actinomyces odontolyticus*.

Additionally, not only that gut microbiota influences host physiological processes, but changes in host immunometabolic processes can alter gut microbiota. Indeed, experimental data suggest that knock out of host-specific signaling for TLR4, interferon-inducible protein AIM2, defensin alpha 5, MUC2, JAMA, apolipoprotein A-I, fatty acid uptake receptor CD36, and aldehyde dehydrogenase 1 family member L1, AHR, vitamin D receptor, etc. results in altered gut microbial profile [235]. Therefore, altered tissue microenvironment in cancer can also influence altered tissue-specific microbial profile. The plethora of current reports demonstrating a correlative-association between cancer-related parameters and tissue-resident microbiota fails to identify whether altered microbiota triggers cancer or remodeling of tissue environment during cancer affects tissue-resident microbiota. Since both conditions are possible, controlled experiments with a reductionist approach are necessary to determine the host-microbiota reciprocal interactions in cancer. Moreover, in the current review and the available literature, the majority of the reports of cancer-related microbiota are from CRC patients and, thus, critically lack understanding of cancer-related host-microbiota interactions at the extraintestinal origin. Several of these studies are also performed using experimental animal models (e.g., germ-free mice) that are already criticized for harboring altered tissue-specific responses and microbial populations than humans [306,307,308].

Collectively, the review has compiled substantial evidence supporting the role of tissue-specific microbes in promoting and in the progression of cancers. Most importantly, the opportunistic association of tissue-resident commensals in promotion and progression of cancers have been highlighted. This essentially highlights the fact that the good, bad, and the ugly characteristics of tissue commensals are conditions specific. These evidences are expected to facilitate microbiota-centered evidence-based anti-cancer strategies through future translational studies.

## Figures and Tables

**Table 1:** Changes in gut microbiota in patients with cancer-causing viral infections.

Infection	Study Population	Study Characteristics	Observed Microbial Changes	Reference
Pulmonary Tuberculosis	31 healthy controls vs. 46 patients from China	Patients with active Mycobacterium tuberculosis infection; gut microbial signatures using shotgun sequencing	Depletion of SCFA producing microbes (*Roseburia inulinivorans*, *R. hominis*, *R. intestinalis*, *Eubacterium rectale*, and *Coprococcus comes*, *Bifidobacterium adolescentis* and *B. longum*, *Ruminococcus obeum*, and *Akkermansia muciniphila*); lower microbial metabolic functions related to SCFA production; decrease in alpha diversity.	[128]
Hepatitis B virus (HBV)	30 Asymptomatic HBV carriers, 31 chronic hepatitis B, 31 decompensated HBV cirrhosis, and 32 health controls from China	16S rRNA sequencing of fecal microbiota and qPCR-based analysis of bacterial virulence genes	Depletion of *Lactobacillus*, *Pediococcus*, *Leuconostoc*, and *Weissella* in symptomatic patients; variation in *F. prausnitzii*, *E. faecalis*, and Enterobacteriaceae in asymptomatic carriers; lower Bifidobacteria-to-Enterobacteriaceae ratio in subjects with infections; lower abundance of *Clostridium* clusters XI and XIVab in decompensated HBV cirrhotic patients.	[129]
Urinary tract infection	168 kidney transplant patients, with 30% developing Enterobacteriaceae bacteremia within 6-mo of transplantation from USA	16S rRNA sequencing; fecal samples	Increased abundance of *Faecalibacterium* and *Romboutsia*, and lower *Lactobacillus*; decreased microbial diversity.	[130]
Human immunodeficiency virus (HIV)	31 HIV patients (18 with antiretroviral treatment) vs. 27 healthy controls from France	16S rRNA sequencing; fecal samples	Lower microbial diversity in HIV patients; Lower *Clostridia*, *Subdoligranulum*, *Ruminococcus*, *Blautia*, *Faecalibacterium*, *Bifidobacterium*, and increased gamma-proteobacteria, Enterococcus in HIV patients; systemic inflammatory markers were inversely correlated with *R. bromii* and *F. prausnitzii*, *whereas associated with E. coli*, *Enterobacter aerogenes*, *E. faecalis and E. faecium*.	[131]
Human papillomavirus (HPV)	345 women having infection with 27 different HPV types Sweden	16S rRNA sequencing; vaginal fluid samples	Prevalence of *Lactobacillus crispatus* and *L. iners*; infected subjects had higher microbial diversity; abundance of *Sneathia*, *Prevotella*, and *Megasphaera* were associated with HPV infection.	[132]
Hepatitis C virus (HCV)	166 HCV infected patients vs. 23 healthy subjects from Japan	16S rRNA sequencing from fecal samples	Less abundance of *Lachnospiraceae* and *Ruminococcaceae* in the patients; a decrease of *Streptococcus salivarius* and increase of *Lactobacillus* spp. with disease progression.	[62]
Chlamydia trachomatis	42 infected and 35 non-infected subjects from Malaysia	16S rRNA sequencing; endocervical swab samples	Lower abundance of *Tenericutes* and Proteobacteria, and increased abundance of *Delftia*, *Streptococcus*, *Pseudomonas*, *Cloacibacterium*, *Prevotella*, *Veillonella*, *Megasphaera*, *Ureaplasma*, and *Ralstonia* in infected subjects	[133]
Opisthorchis viverrini	30 infected and 26 non-infected subjects from Russia; all 54 were diagnosed with cholelithiasis	16S rRNA sequencing of samples from gall bladder	Increased abundance of Spirochaetes, Planctomycetes, Synergistetes, Verrucomicrobia, and Saccharibacteria (TM7) in infected patients; detection of *Veillonella dispar*, *Paracoccus aminovorans*, *Parabacteroides distasonis*, *Sphingomonas changbaiensis*, *Cellulosimicrobium* sp., *Phycicoccus* spp. only in infected patients, whereas *Flectobacillus* sp., *Xanthobacter* sp., *Burkholderia* sp., *Streptomyces* sp., *Jeotgalicoccus psychrophilus*, and *Treponema socranskii* present only in un-infected subjects.	[134]
Urogenital schistosomiasis	116 pre-school children with infection from UK	16S rRNA sequencing from fecal samples	The most abundant genera were *Prevotella*, *Bacteroides*, *Alistipes*, *Eubacterium*, *Faecalibacterium*, *Clostridium*, *Roseburia*; *Pseudomonas*, *Azospirillum*, *Stenotrophomonas*, *Derxia*, and *Thalassospira* were associated with infection.	[135]
Kaposi’s sarcoma (KS)-associated herpesvirus	29 subjects from USA with pathology-confirmed KS who were serologically positive for KS-associated herpesvirusand HIV infection	16S rRNA gene sequencing of samples from an oral swab	Lower microbial diversity and observed species and distinctly altered microbial taxonomic signatures in subjects with oral KS without any oral cell-associated HIV infection; the abundance of *Aggregibacter* and *Lautropia* were higher, but *Corynebacterium* and *Shuttleworthia* were lower in subjects with no oral KS.	[136]

**Table 2:** Microbiota centric strategies against cancer using prebiotics.

Study Characteristic	Observations	References
Dietary fiber intake was assessed in patients diagnosed with advanced colorectal adenoma to colorectal cancer (n = 344) and healthy controls (n = 47) from China.	Patients had reduced dietary fiber-intake patterns and consistently decreased SCFA production, less prevalence of *Clostridium*, *Roseburia*, and *Eubacterium* spp, and low abundance of *Enterococcus* and *Streptococcus* spp.; Fecal butyrate levels and butyrate-producing bacteria were high in a subset of cancer patients with comparatively higher fiber intake.	[244]
A meta-analysis of 24 studies to define how effective dietary fiber consumption is at lowering the risk of breast cancer	Dietary fiber consumption was found to reduce the incidence of breast cancer by 12%; Based on the type of studies and menopausal status, the link between dietary fiber consumption and breast cancer risk was substantial. A dose-response study revealed that every 10 g/d increase in dietary fiber consumption was linked to a 4% reduction in the incidence of breast cancer.	[245]
Dietary questionary-based examination of 519,978 people (25–70 y age) to link dietary fiber intake with colorectal cancer incidence in Europe.	The amount of dietary fiber in meals was inversely associated with the occurrence of large bowel cancer; However, there was no evidence that one kind of fiber was significantly more protective than another.	[246]
The association of dietary fiber intake with colon and rectal cancer was assessed in 1168 cancer patients out of a cohort of 108,081 persons from the Scandinavian population	There was an inverse relationship between total fiber intake and the risk of colon cancer with each additional increase of 10 g/d and 2 g/d fiber consumption for males and females, respectively.	[247]
Investigation of the links between whole grain and dietary fiber consumption and the risk of liver cancer and death from chronic liver disease in 485,717 subjects from the USA.	Higher grain intake was linked to a decreased incidence of liver cancer and death from chronic liver disease; Dietary fiber was also linked to a reduced incidence of liver cancer.	[248]
Dietary questionary-based analysis of the association of fiber intake with renal cancer risk in 491,841 subjects in the USA.	Total dietary fiber consumption was linked to a 16–20% decreased incidence of kidney carcinoma; The negative relationship between fiber consumption and renal cancer was seen in people who had never smoked, had a low BMI and had no history of diabetes or hypertension.	[249]
A meta-analysis of 24 studies (580,064 subjects) was performed to study the effects of dietary fiber consumption on the risk of gastric cancer.	Dietary fiber consumption is linked to a lower risk of gastric cancer, and this impact is likely independent of other risk variables; A dose-response study found that increasing fiber consumption by 10 g per day reduced the incidence of stomach cancer by 44%.	[250]
Dietary fiber intake was evaluated in a US-based cohort study, including 463 head and neck cancer patients.	Increasing dietary fiber consumption before starting treatment can help patients longer; No statistically significant links between whole grains and prognostic outcomes were identified.	[251]
A dose-response meta-analysis of dietary fiber intake in 13 studies (142,189 participants) consisting of 5777 ovarian cancer patients	Dietary fiber intake and the risk of ovarian cancer have a substantial inverse dose-response relationship.	[252]

**Table 3:** Microbiota centric strategies against cancer using probiotics.

Study Characteristic	Observations	References
Milk fermented by *Lactobacillus casei* CRL 431 supplementation for 80-d. Female BALB/c mice of 6-wk age were challenged with 4T1 breast cancer cells.	Attenuated tumor growth, vasculature, extravasation, and metastasis; lower macrophage infiltration within the tumor microenvironment and lungs; increased CD8+ T-cell mediated tumor cytotoxicity; improved CD4+ T-cell populations.	[269]
*Lactobacillus plantarum* YYC-3 isolated from fermented rose. C57BL/6-APC^Min/+^ mice with colon cancer supplemented with a high-fat diet were treated with either 10^9^ CFU of *L. plantarum* YYC-3 or cell-free bacterial supernatant.	Reduced mucosal injury and tumor incidents; lowered the populations of inflammatory lymphocytes and the levels of IL-6, IL-17, IL-22; attenuated NFκB activation and Wnt signaling pathway; restored altered microbiota composition with increased abundance of gut commensals.	[270]
*Lactobacillus reuteri GMNL-89* and *Lactobacillus paracasei GMNL-133* in 1:1 ratio administered 5-d per wk for 4 wk. Mice models of pancreatic cancer (LSL-K-rasG12D; Pdx-1-cre) were orally treated with 10^9^ viable *P. gingivalis*	Decrease body weight; tissue expression of Snail-1, ZEB-1, collagen fibers, Galectin-3, and PD-L1 were attenuated; attenuated expression of total Smad3 and phosphorylated Smad3; Reduced cancer cell proliferation, viability, pancreatic intraepithelial neoplasia, and metastasis likely by affecting transforming growth factor-β signaling pathway.	[271]
Supplementation of 10^8^ CFU/d of *Lactobacillus rhamnosus* R0011 to NMRI inbreed mice for 2-wk, followed by grafting of human gastric cancer tissue, then 4-wk treatment of *L. rhamnosus.*	Resulted in tumor regression; an increase in WBC populations, Bax/BCL2 ratio; inflammatory reaction around tumor tissue, cell necrosis, and apoptosis are increased.	[272]
Female BALB/C mice were pre-treated for 14-d with 10^8^ CFU *Lactobacillus acidophilus* NCFM followed by inoculation of CT-26 colon carcinoma cells.	Suppression of tumor growth; reduction of micro-tumor size and initiation of apoptosis in tumor cells; Bcl2 expression was lower whereas caspase-9 and caspase-3 expressions were higher; CXCR4 and MHC class I expression colon and mesenteric lymph nodes.	[273]
*Lactobacillus casei KK378* at 10^4^–10^8^ CFU was injected into tumor site in male BALB/cSlc-nu/nu mice inoculated with head and neck squamous cell carcinoma cell (SAS, HSC2, and HSQ89)	Regression of tumor size; increase levels of TNF-α, IFN-γ, IL-5, IL-10, and IL-12.	[274]
*Lactobacillus salivarius* REN was supplemented at 10^5^–10^10^ CFU per day for 32 wk or 23 wk to Male F344 rats treated for 8-wk with 4-nitroquioline 1-oxide to induce oral cancer.	Reduced incidence of tongue tumors and preneoplastic lesions; decreased level of 8-hydroxydeoxyguanosine (a marker of oxidative stress) in the tongue mucosa; lower expression of COX-2 and proliferating cell nuclear antigen; Degradation of carcinogen.	[275]
C57BL/6 mice were orally supplemented with 2 × 10^8^ CFU of *Lactobacillus rhamnosus* GG for 2-wk	Lowered colonic tumor counts; Increased expression of IL-2, IFN-γ, CXCL9, CXCL10; increased population of CD8+ CD3+ T-cell, granzyme B+ CD8 T-cells, dendritic cells.	[276]
*Lactobacillus acidophilus* cell lysate + anti-CTL antigen-4 blocking antibody treatment for 34-d	Prevents loss of body weight and development of colorectal cancer; tumor microenvironment had higher CD8+ T cells, CD44+ CD8+ CD62L+ effector T-cells, and lower CD4+ CD25+ Foxp3+ T-reg and F4/80+ CD206+ M2 macrophages; reduced macrophage M2 polarization and IL-10 expression in LPS-treated macrophage; reduce the fecal abundance of proteobacteria.	[277]
Supplementation of *Bifidobacterium lactis* at 10^11^ CFU/g with or without resistant starch (100 g/kg diet) for 22-wk to Sparge-Dawley rats treated with azoxymethane for induction of colon cancer.	Reduction of frequency and development of colonic neoplasm; increased SCFA production; increased crypt column length, and decreased PCNA+ cells.	[278]

## Data Availability

No experimental data are associated with this paper.

## Abbreviations

4-HNE, 4-hydroxy-2-nonenal; A-T, Ataxia-telangiectasia; AID, Activation-induced cytidine deaminase; ALL, lymphoblastic leukemia; ASIR, age-standardized incidence rate; Avr A, Avirulence protein A; BFT, B. fragilis toxin; CAC, colitis-associated cancers; CNF, cytotoxic necrotizing factor 1; CR, calorie restriction; CRC, Colorectal cancer; EBV, Epstein-Barr virus; EPEC, enteropathogenic *E. coli*; F:B, Firmicutes-to-Bacteroidetes ratio; FMT, fecal microbiota transplantation; GC, gastric cancer; GI, gastrointestinal; HBV, hepatitis B virus; HCC, Hepatocellular carcinoma; HCV, hepatitis C virus; HHV8, Human Herpesvirus 8; HIV, Human immunodeficiency virus; HPV, Human Papillomaviruses; KRAS, Kirsten ras; KS, Kaposi’s sarcoma; MDR, Multidrug-resistant; MMR, Mismatch repair; PDAC, pancreas ductal adenocarcinoma; PMT, Pasteurella multocida toxin; SCFA, short-chain fatty acid; SIBO, small-intestinal bacteria overgrowth; TGF-beta, transforming growth factor-β1; TNF, tumor necrosis factor.
